# Modelling the Dynamics of an Experimental Host-Pathogen Microcosm within a Hierarchical Bayesian Framework

**DOI:** 10.1371/journal.pone.0069775

**Published:** 2013-08-02

**Authors:** David Lunn, Robert J. B. Goudie, Chen Wei, Oliver Kaltz, Olivier Restif

**Affiliations:** 1 Medical Research Council Biostatistics Unit, Institute of Public Health, Cambridge, United Kingdom; 2 Institut des Sciences de l'Evolution, CNRS UMR 5554, Université Montpellier 2, 34095 Montpellier, France; 3 Disease Dynamics Unit, Department of Veterinary Medicine, University of Cambridge, Cambridge, United Kingdom; Albert Einstein College of Medicine, United States of America

## Abstract

The advantages of Bayesian statistical approaches, such as flexibility and the ability to acknowledge uncertainty in all parameters, have made them the prevailing method for analysing the spread of infectious diseases in human or animal populations. We introduce a Bayesian approach to experimental host-pathogen systems that shares these attractive features. Since uncertainty in all parameters is acknowledged, existing information can be accounted for through prior distributions, rather than through fixing some parameter values. The non-linear dynamics, multi-factorial design, multiple measurements of responses over time and sampling error that are typical features of experimental host-pathogen systems can also be naturally incorporated. We analyse the dynamics of the free-living protozoan *Paramecium caudatum* and its specialist bacterial parasite *Holospora undulata*. Our analysis provides strong evidence for a saturable infection function, and we were able to reproduce the two waves of infection apparent in the data by separating the initial inoculum from the parasites released after the first cycle of infection. In addition, the parameter estimates from the hierarchical model can be combined to infer variations in the parasite's basic reproductive ratio across experimental groups, enabling us to make predictions about the effect of resources and host genotype on the ability of the parasite to spread. Even though the high level of variability between replicates limited the resolution of the results, this Bayesian framework has strong potential to be used more widely in experimental ecology.

## Introduction

The last two decades have seen unprecedented progress in statistical modelling of epidemic dynamics [1]. However, to our knowledge these techniques have not been much used in experimental host-parasite systems, despite their great potential. Host-pathogen model systems maintained in the laboratory have for many years played an important role in the study of the fundamental processes driving the spread of infections in populations [2], [3]. In particular, the validity of mass action to model disease transmission has been assessed in various systems, with differing results, but often in isolation from other processes [4], [5]. In systems where other processes such as death, recovery or multiple routes of transmission are important, horizontal transmission cannot be assessed on its own; instead it is necessary to fit a complete dynamic model to observed time series [6]. In experimental systems, such an integrative approach would allow quantitative assessment of the effects of various ecological or genetic factors on transmission rates, and would provide the basis for developing system-specific models for the evolution of hosts and pathogens.

Despite substantial research efforts in this field over the last three decades, there is still a wide gap between theoretical and experimental studies of host-pathogen evolution: mathematical models have been developed for many years using *ad hoc* assumptions about the potential trade-offs faced by hosts or pathogens. On the pathogen side, it is often assumed that there is a positive relation between infectivity and virulence [7]. On the host side, resistance is thought to come at a cost (e.g. survival, fecundity or tolerance) [8]. Interestingly, both cases involve one trait that can be measured at the individual level (survival or fecundity), and one that involves transmission and thus must be measured at the population level. It is therefore essential to be able to relate experimental measures at the individual and population levels, as we will show is possible in our framework.

We seek in this study to tease apart the main components of the infection cycle in an experimental host-parasite system, and investigate how environmental and host genetic factors quantitatively affect these dynamics. The biological system that we examine is composed of the free-living protozoan *Paramecium caudatum* and its specialist bacterial parasite *Holospora undulata* [9]. The parasite has a complex infection cycle that alternates between two stages that have different horizontal and vertical transmission abilities and different levels of virulence [10], [11]. The complexity makes this system a good candidate for an integrative modelling approach because it is very difficult to isolate experimentally the different processes involved. Our study associates three main elements: experimental time series data, a mathematical model that describes the dynamics of the system, and a Bayesian statistical framework that fits the dynamic model to the data and characterises the different sources of variability.

An important feature of this approach is our use of information gathered from separate experiments. Traditionally, such information is translated into fixed values for some parameters of the model, whilst the remaining parameters are estimated from the new data. Here we use a Bayesian framework, in which parameters have probability distributions reflecting the uncertainty about their values: the *prior* distribution encapsulates what is known about the parameters *before* seeing (or independently of) the data currently of interest, whereas the *posterior* distribution reflects what is known *after* taking the data into account. We show how readily this approach can be applied to experimental ecology and can accommodate multi-factorial experimental design, multiple response variables measured over time, sampling error and non-linear dynamics. Importantly, our approach highlights how much information is available from a dataset in relation to a mechanistic model, and allows comparison between alternative models. Bayesian inference has become the method of choice for fitting dynamic infection models to time series of case reports [12], and several authors have explored its use for inference for differential equations [13], including in the context of experimental infection dynamics [14]. Our work focuses on the hierarchical structure of the model, extending the work of Mideo *et al.* [15], and further demonstrates the feasibility and flexibility of Bayesian models for experimental ecology.

The hierarchical structure of the statistical model enables us to collate evidence efficiently about parameters from all of the available data into a unified model, allowing the degree of similarity between treatment regimes to be assessed. Estimating the variability across various levels of the hierarchy also naturally provides both population and treatment-level estimates of means and variances, enabling detection of significant differences between treatments [16]. Predictions about future treatments can also be made straightforwardly. Finally, hierarchical models supplement information on each replicate with information from other replicates via their joint effect on the estimated population parameters. This effect is known as ‘borrowing of strength’ [17] and is particularly useful in settings where only limited replicate-level data are available, as here. Tying together the analysis of the different treatment regimes into an overall hierarchical model thus uses the available data more efficiently and is more informative than analysing each regime separately.

## Methods

### 2.1 Experimental system

#### 2.1.1 Study organisms

The biological system used consists of the ciliate protozoan *Paramecium caudatum* (Ehrenberg 1833) and its specialist bacterial parasite *Holospora undulata* (Hafkine 1890). We have developed this system for several years as an experimental model for investigating host-parasite ecological and evolutionary dynamics [10], [18], [19]. Paramecia are free-living planktonic protozoa, commonly found in fresh water ponds, that feed on a wide range of bacteria. *H. undulata* is a Gram-negative *α*-proteobacterium that colonises the micronucleus of *P. caudatum*. At some point it produces non-dividing, spore-like particles, which are released into the environment following the division or the death of host cells and can infect new paramecia. We refer to these particles as infectious forms, and to the intracellular replicating stage as reproductive forms. The two forms can be easily distinguished under the microscope as the reproductive forms are rod-shaped whereas the infectious forms have an elongated S-shape. Both forms can be passed on to the progeny of an infected paramecium when it undergoes clonal division (vertical transmission), but only the infectious forms can infect a new host cell following their ingestion (horizontal transmission).

#### 2.1.2 Experimental setup

A total of 48 experimental populations of *P. caudatum* were grown and monitored in parallel over 34 days, following a full factorial design with three factors: *P. caudatum* clone (K4, K6, K8 and K9), food concentration (high or low) and inoculum (infected with the parasite or uninfected control). Each combination of treatments was replicated in three populations. The four *P. caudatum* clones were full-sibs derived from the conjugation of two parental clones with complementary mating types O3 and E3 (provided by T. Wanabe, Tohoku University, Japan). Bacterial food for paramecia consisted of *Serratia marcenscens* (Institut Pasteur, Paris) in a suspension of Protozoan Pellets (Carolina Biological Supply Company, Burlington, NC) in Volvic mineral water. The high food treatment had a concentration of 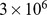 bacteria per ml and 0.7 mg of food pellets per ml; the low food treatment was obtained by a 50% dilution of the former in mineral water. The parasite inoculum of *H. undulata* was isolated from a *P. caudatum* population provided by H. Görtz (University of Stuttgart, Germany); infected paramecia were ground mechanically and the released parasites concentrated to 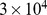 infectious forms per ml. Further details of the experimental setup and techniques are given elsewhere [11]. The mock inoculum was obtained by the same procedure starting from an uninfected *P. caudatum* stock.

Each population initially consisted of, on average, 500 uninfected paramecia, to which we added 0.1 ml of either *H. undulata* inoculum (for the infected half of the populations) or mock-inoculum (for the other, non-infected half of the populations). We started the experiment with small population sizes so that within a few weeks the paramecia populations would have grown to their carrying capacity, which, based on preliminary experiments, we expected to be around 5000 paramecia with the high food treatment and around 2500 paramecia in the low food treatment. Every population was sampled twice a week (totalling 11 time points) to assess the number of paramecia and their infection status (using DNA staining and optical microscopy). Every sampling event of each population removed a small enough number of paramecia (between 20 and 40) that their removal can be ignored in our population dynamic model.

We measured the division rate, survival rate and infection status on days 3 and 31 by isolating eight paramecia from each population and keeping them in separate drops of culture medium for between two to three days [11]. The results from these two sub-experiments, in combination with other evidence [10], [11], [20], guided the choice of prior distributions for the model parameters, as explained below.

### 2.2 Mechanistic model

#### 2.2.1 Basic model

**Figure 1:**
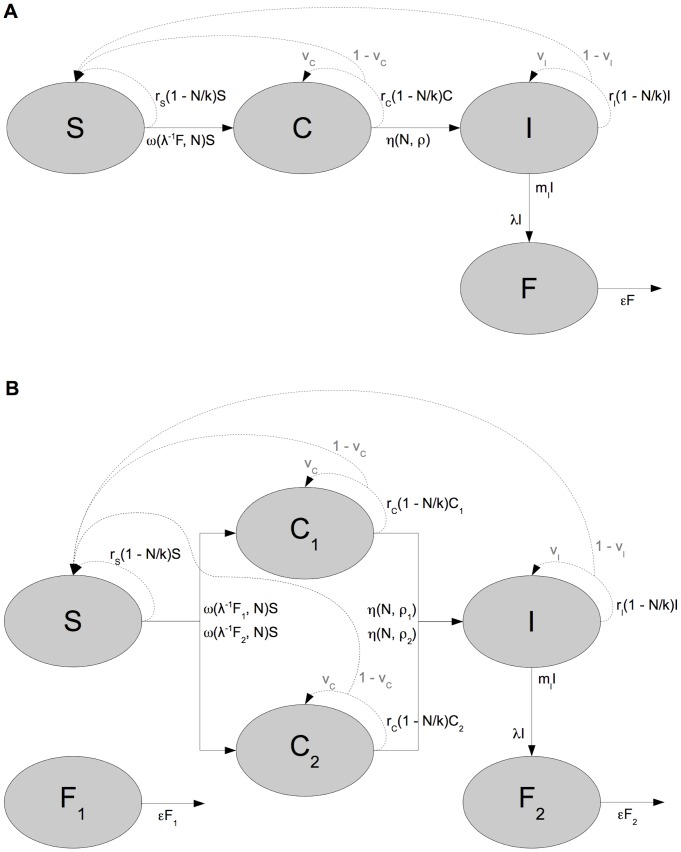
Compartmental models. (a) without distinction between the inoculum and newly-produced parasites, (b) with distinction.

We designed a mathematical model, illustrated by the flow chart diagram in Figure 1A, to capture the population dynamics of our system. Our aim is to demonstrate the feasibility of our method for experimental host-pathogen systems and so we work with a system of ordinary differential equations, which is the most commonly used modelling framework in ecology and epidemiology. Although *H. undulata* replicates within paramecia, we were not able to estimate bacterial loads experimentally. We thus categorise infected paramecia as carriers (*C*) if they only harbour reproductive forms of the parasite, and as infectious (*I*) if they harbour a mixture of reproductive and infectious forms. Susceptible paramecia (*S*) become carriers following ingestion of infectious forms (*F*), which we assume are released by infectious paramecia into the medium at a constant rate. We denote by *C*, *I*, *S* and *F* the number of organisms of those types, and let 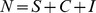 denote the paramecium population size.

Paramecia divide clonally and we assume populations follow a logistic growth model with carrying capacity *k* (equilibrium population size in the absence of infection). Each host-type 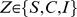 reproduces at a rate 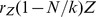. Upon division, infected paramecia pass on their infected status (*C* or *I*) to a fraction (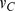 or 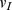) of their progeny; we refer to this process as imperfect vertical transmission of the parasite. In line with separate observations [11], we assume that paramecia containing infectious forms of the parasite suffer from an additional death rate 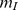. Free parasites are released from infectious host cells either during host division or following their death; for simplicity we modelled this release as a continuous process with rate 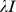. The parasite population decays at a rate 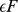. There are no observations within our data regarding the size of the parasite population *F* and so it is not possible to identify the scale *λ*. We thus express the model in terms of 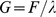.

One of our objectives is to clarify the functional form of the infection process, which occurs via grazing of non-motile bacteria by motile paramecia. We consider three alternative forms for the force of infection 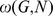: classical mass action, 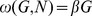; a *G*-saturating function, 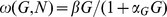, which accounts for the fact that paramecia can ingest only a limited number of bacteria per unit of time; or an *N*-saturating function, 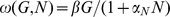, as might occur for example if only a limited number of paramecia could predate on the bacteria because of restrictions in space.

It has been reported [10] that infectious forms of *H. undulata* are produced from reproductive forms inside infected paramecia more rapidly in more dense populations (i.e. closer to carrying capacity). We therefore assume that the rate of conversion *η* of carrier hosts *C* into infectious ones *I* increases linearly with population size *N*:
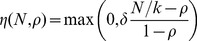
(1)The conversion rate is zero until the total population *N*, as a proportion of the carrying capacity *k*, reaches a threshold *ρ*. For larger *N*, the rate increases linearly and is equal to *δ* when 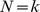. This reflects a time delay between hosts becoming infected and maturing into infectious forms. An initial period during which no infectious forms are created has been observed experimentally [21].

#### 2.2.2 Two-wave model

Pilot experiments showed two successive peaks in the number of infected hosts, which we hypothesised to be the result of the delay in production of infectious forms. Numerical exploration of the basic model indicated that it could produce only one wave of infection. This suggested an extended model in which free infectious forms 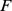 are sub-divided between the inoculum, 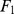, and the bacteria produced and released by infected paramecia, 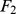. In order to allow a delay in the production of infectious forms which generates the second wave, we also partition the ‘carrier’ compartment 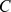 into 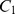, the population of hosts infected by 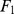, and 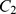, the population of hosts infected by 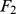. Conversion from carrier to infectious occurs at rate 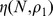 from 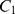 to 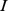, and at rate 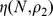 from 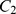 to 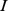. As above, we use 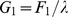 and 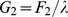 to express our model because we have no observations of the size of the parasite population. Where relevant, we let 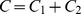 and 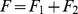. The system is described by differential equations (2)–(7) below, and is shown as a flow diagram in Figure 1B.
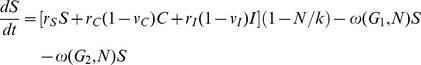
(2)

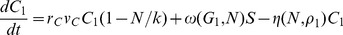
(3)

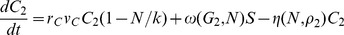
(4)

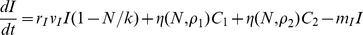
(5)

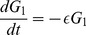
(6)

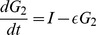
(7)


### 2.3 Statistical model

#### 2.3.1 Observation model

Let 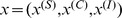 denote the observed number of host cells in each category in the sampled population. Further, let 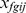 denote the 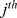 sampled population, measured at time 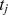, for replicate *i* of genotype *g* at food level *f* (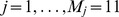, 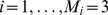, 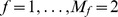, 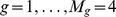). Denoting the observed total number of host cells in the sampled population by 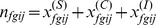, the sampled host-populations 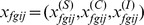 are modelled via:
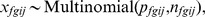


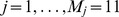
, 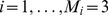, 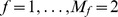, 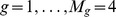, where 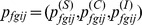 denotes the modelled proportion in each state:
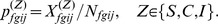
Here 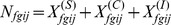, and 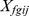 = 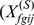, 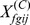, 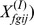 is the modelled total population in each state, given by solving (2)–(7) at time 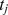, with replicate-specific parameter vectors 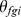 (see below) and appropriate initial conditions, denoted 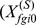, 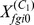, 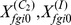, 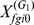, 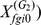. The initial values (just after inoculation) for 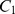, 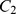, 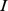 and 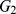 are known to be zero, whereas 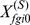 and 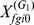 are unknown and are assigned appropriate prior distributions, as described in Section 2.3.3.

Inference on the total host-populations is driven by relating the observed total number of host cells in the sampled population 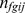 to the modelled total population in each state 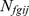. We might consider the following assumption: 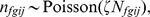 where 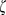 is the proportion of the total volume sampled to obtain each 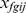. However, this (implicitly) assumes that the system is entirely homogenous and that the modelled total population 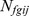 is exact. Realistically, the model for the total population (e.g. (2)–(7)) is an approximation of reality, and so it is appropriate to allow for some error:
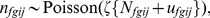
(8)where 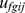 represents the difference between 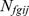 and the *true* total population, and might be assumed to arise from a normal distribution with zero mean and unknown variance (to be estimated). Implementation of this model requires estimation of each individual 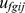 term, and the model remains reliant on an assumption of homogeneity. One alternative is to assume the 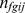
s arise from a negative binomial distribution, (see e.g., [17], pp. 116–118) but this is cumbersome to implement and estimation can be slow. We have chosen to use a simpler alternative, which has given virtually identical results to (8) for the data considered herein:
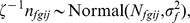
where 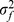 is a food-level-specific, unknown variance parameter.

#### 2.3.2 Model parameterisation

The mechanistic model contains 13 unknown parameters: 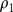, 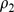, 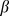, 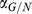, 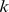, 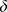, 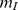, 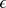, 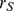, 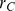, 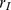, 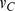 and 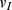, where 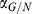 represents either 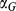 or 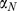 as appropriate. We will specify our prior knowledge regarding each parameter and express the variation across different replicates and genotypes in terms of a parameter vector 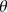. We define 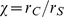 and 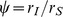 because our prior beliefs regarding the reproduction rates for carriers and infectious hosts (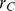 and 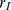 respectively) are more readily expressed in terms of fractions of the rate 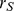 for susceptible hosts. We also define 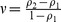, which we use to impose the constraint 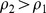 on the thresholds for production of infectious parasites for the first and second waves, as discussed below. It was decided to exclude the fidelities of vertical transmission 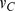 and 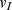 from the set of unknown parameters and we fix these parameters equal to 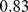 and 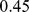 respectively, based on direct experimental measurement [11]; preliminary analyses of the model showed that the values of these two parameters have little effect on the dynamics, and their inclusion in the set of unknown parameters would likely result in identifiability issues. This leads to the following vector of 11 unknown parameters:
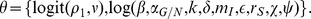
The transformations applied to the parameters enforce positivity of all parameters, and ensure that 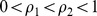.

#### 2.3.3 Parameter model

Our model estimates a distinct set of parameters, denoted 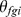, for each replicate *i* from each genotype *g* at each food level *f*, but we expect that experimental populations in the factorial design of the experiment under an identical regime of treatments are more similar than those with differing treatments. We also expect similiarities between experimental populations that share only part of their treatment regime. These assumptions can be encapsulated by a *Bayesian hierarchical model* in which experimental populations are first grouped according to food level and then by genotype. We construct a hierarchical model for the corresponding parameters 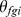 for each experimental population. Specifically we assume that the parameters 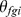 across replicates 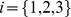 for a given food level 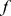 and genotype 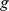 are similar, and so we assume they are drawn from a common distribution:
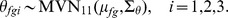
Here 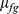 denotes a set of unknown mean parameters for genotype 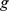 at food level 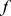, and 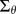 denotes the unknown (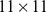) covariance of parameters across replicates.

Similarly, we assume that the parameters 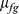 for a given food level 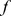 are alike and so are assumed to be drawn from a distribution that is common to experimental populations with the same food level 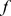. Specifically, we assume that
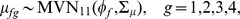
where 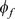 comprises unknown *global mean* parameters for food level 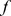, and 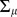 denotes the unknown (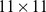) covariance of genotype-specific means across genotypes.

Our prior beliefs for the initial conditions 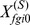 and 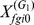, and the parameters 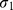, 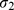, 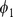, 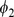, 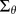 and 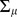 are shown in Table 1; full details of the prior distributions assigned are given in Text S1 in File S1.

**Table 1:** Symbols and summaries of prior information for variables used in this study.

	Definition	Prior range	Prior belief	Source
*S*	Number of susceptible hosts	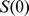 in 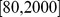	0.95	Study design
*C*_1_	Number of carrier hosts infected by the inoculum	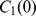 set to 0	—	Study design
*C*_2_	Number of carrier hosts infected by newly-produced parasites	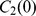 set to 0	—	Study design
*I*	Number of infectious hosts infected by the inoculum	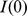 set to 0	—	Study design
*F*_1_	Number of free parasites from the inoculum	—	—	
*F*_2_	Number of newly-produced free parasites	—	—	
*G*_1_	Re-scaled variable: 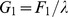	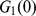 in 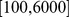	0.95	Study design
*G*_2_	Re-scaled variable: 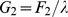	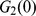 set to 0	—	Study design
*r_S_*	Replication rate of *S* (day^−1^)	LF 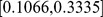	0.95	Control analysis
		HF 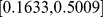	0.95	Control analysis
*r_C_*	Replication rate of *C* (day^−1^)	—	—	
*χ*	Relative rate: 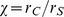	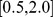	0.95	[11]
*r_I_*	Replication rate of *I* (day^−1^)	—	—	
*ψ*	Relative rate: 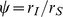	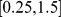	0.8	[11]
*k*	Carrying capacity	LF 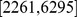	0.95	Control analysis
		HF 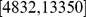	0.95	Control analysis
*m_I_*	Additional death rate of infectious hosts (day^−1^)	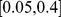	0.67	[11]
*ρ*_1_	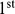 wave threshold for production of infectious parasites	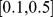	0.95	[10]
*ρ*_2_	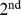 wave threshold for production of infectious parasites	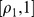	1	[10]
*β*	Infection rate			
(day^−2^)	Vague	—	—	
*α_N_*	Factor controlling saturation of infection	[ 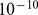 , 0.125]	0.95	—
*α_G_*	Factor controlling saturation of infection	[ 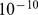 , 0.1]	0.95	—
*δ*	Maximum conversion rate from *C* to *I* (day^−1^)	Vague	—	[10]
*ε*	Decay rate of free parasites (day^−1^)	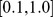	0.5	[20]
*v_C_*	Fidelity of vertical transmission for carriers	Set to 0.83	—	[11]
*v_I_*	Fidelity of vertical transmission for infectious hosts	Set to 0.45	—	[11]

LF: low food, HF: high food. See Text S1 in File S1 for an explanation of how prior distributions were obtained.

#### 2.3.4 Identifiability issues

When fitting a full hierarchical model for *θ* we found that the parameter estimates for the relative rates of reproduction *χ* and *ψ* were widely divergent from our prior beliefs. We investigated various approaches for addressing this issue, and chose the following attractively straightforward and pragmatic solution (please see Text S2 in File S1 for further discussion). In this approach, we assume that for each replicate, within each genotype, the relative rates of reproduction *χ* and *ψ* have marginally independent, as opposed to conditionally independent (given some unknown mean and variance), priors:
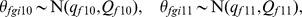
where 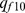 and 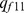 are the prior means, and 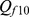 and 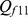 are the prior variances of 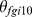 and 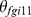, which denote the 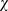 and 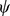 components of the parameter vector 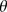, respectively.

### 2.4 Inference

We approximate the posterior distribution of our model using Markov chain Monte Carlo (MCMC) methods [22], using the freely available WinBUGS software [23], [24], with the system of differential equations (2)–(7) specified via the *WBDiff* interface (www.winbugs-development.org.uk). Using the posterior distribution, we can formally compare the parameters for different food levels and genotypes. We describe differences as ‘significant’ if the 95% credible interval for the difference between the relevant parameters does not include 0. For example, we examine the 95% credible intervals of the set of contrasts 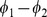, between the mean parameters of the two food levels.

## Results

### 3.1 Experimental populations

**Figure 2:**
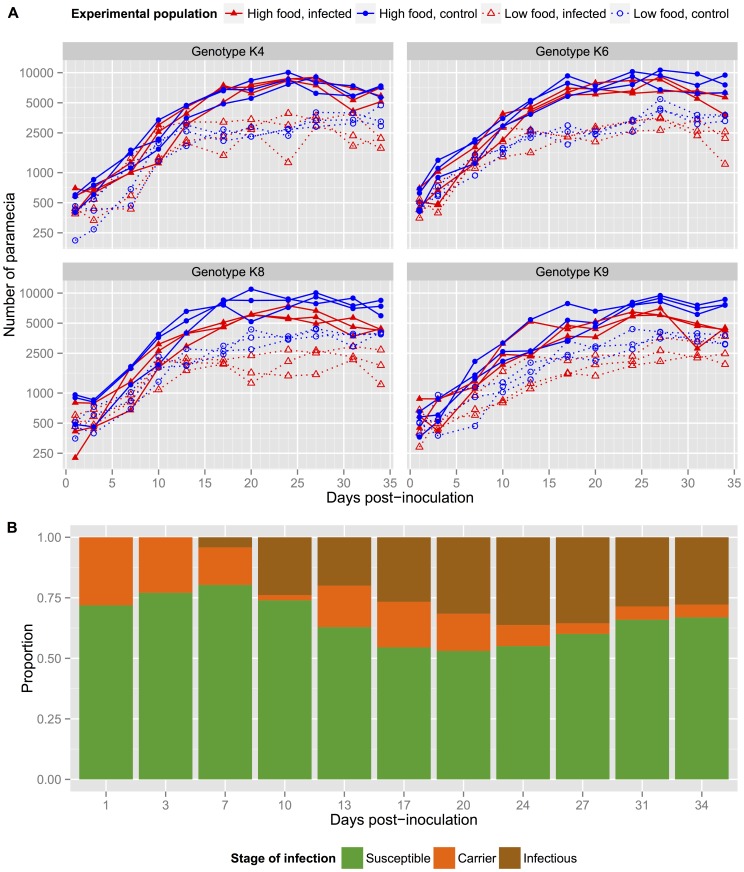
Experimental data. (a) Time series of the number of paramecia in each of the 12 populations of each clone, classified by inoculum and food level treatments; note the logarithmic scale. (b) Time series of the mean proportions of paramecia in each of the three stages of infection (green: S, amber: C, brown: I) across all populations.

The observed dynamics were highly variable between populations (Figure 2A). Broadly speaking, every population appeared to exhibit logistic growth, although some tailed down over the final week or so. Across all combinations of paramecium clones and inoculum treatments, populations in low food reached lower densities than their counterparts in high food level (two to four fold differences). In addition the data suggest a slight negative effect of infection in clones K8 and K9 only (Figure 2A).

The dynamics of infection appear to follow three main stages (Figure 2B): initially infected paramecia contain only reproductive forms, until the appearance of infectious forms around day 7 or 10; then a second, transient wave of reproductive forms (carriers) appear around day 13–17; eventually the non-susceptible population is dominated by infectious forms for the last 10–14 days. These patterns appeared to be consistent with two waves of infection, one caused by the initial inoculum and a subsequent one caused by the release of infectious forms by the first cohort of infected paramecia.

### 3.2 Model selection

Our first objective was to compare a set of alternative model structures based on their goodness of fit to the dynamics of each experimental population and their ability to reproduce qualitatively the main features highlighted above. More specifically, we sought to clarify two aspects of the host-pathogen dynamics: first, whether the force of infection 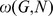 is best described by mass action or by a saturable function; second, whether separate variables for inoculum and newly-produced infectious bacteria helps to reproduce the two waves of infection (see section 2.2). We thus compared six different models, obtained by combining each of the three alternative forms for the force of infection *ω* (mass-action, *G*-saturating or *N*-saturating) with either the one-wave or two-wave modelling framework.

In each case, one million posterior samples were generated following convergence of the MCMC simulation. Convergence was assessed by running two Markov chains starting from widely differing initial values, and by then applying the Brooks-Gelman-Rubin diagnostic [25], [26]; we also assessed convergence informally by visually examining chain-history plots [17, pp. 71–74] as illustrated in Figure S1 in File S1. Use of multiple chains also helps in confirming that the simulation is not getting trapped in local posterior maxima. To reduce the amount of memory required we “thinned” the samples by saving only every 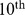 iteration. We were not able to fit the one-wave, *N*-saturating model because the MCMC sampler frequently visited implausible regions of parameter space in which the Runge-Kutta [27] numerical differential equation solver failed to find accurate solutions.

Table 2 shows for each model the posterior mean deviance, which is a measure of model fit [28], with lower values indicating a better fit to the observed data. In hierarchical models the deviance is defined as minus twice the natural logarithm of the joint probability density of the observed data, according to their assumed sampling distributions (BUGS computes this automatically). There is a clear preference for two-wave models, and also for the G-saturating function to describe the rate of infection, regardless of how many waves of infection are assumed. As measured by posterior mean deviance, the two-wave 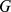-saturating model is the most suitable of the models we considered.

**Table 2:** Posterior mean deviance 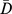 of the six models considered.

Number of waves	Infection function	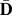
1	Mass action	6816.3
1	*N*-saturating	(*)
1	*G*-saturating	6800.2
2	Mass action	6667.8
2	*N*-saturating	6582.0
2	*G*-saturating	**6544.4**

(*) We were not able to fit the one-wave, *N*-saturating model.

**Figure 3:**
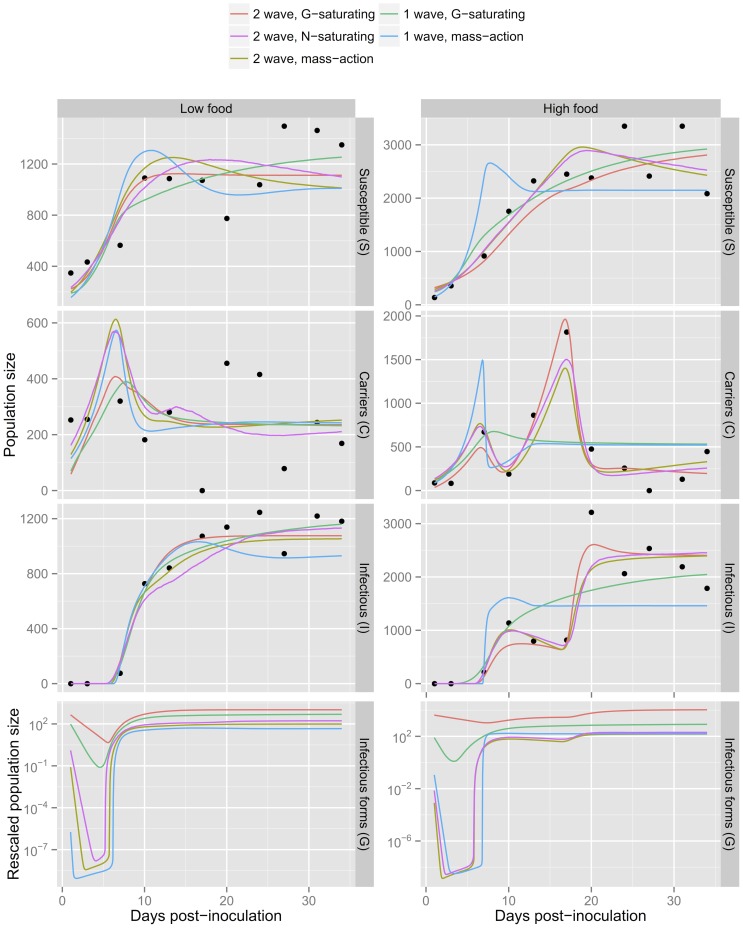
Model fits for clone K8, replicate A. Each panel shows a different variable (from top to bottom: *S*, *C*, *I* and *G*) in low food (left) and high food (right). The dots show experimental data and the lines show the predicted dynamics obtained from each of the five fitted models.

The models can be assessed qualitatively by visually comparing the predicted dynamics with the observations (Figure 3; and Figure S2 in File S1). The one-wave models fail to account for key features of the dynamics: the peak in the number of carrier paramecia (*C*) after around 20 days is entirely missed by the *G*-saturating model, and the mass-action model fails to reproduce the dynamics of both uninfected (*S*) and infectious (*I*) paramecia in the high-food population. The dynamics of the fitted two-wave models are more consistent with the observed time series. In particular, the observations suggest that the magnitude of the second wave of infection (values of *C* and *I*) is much larger than the first wave of infection in most replicates. While all the fitted models tend to underestimate the magnitude of the second wave of infection (Figure 3; and Figure S2 in File S1), the observed difference in magnitude between the waves is reproduced most closely by the *G*-saturating model.

**Figure 4:**
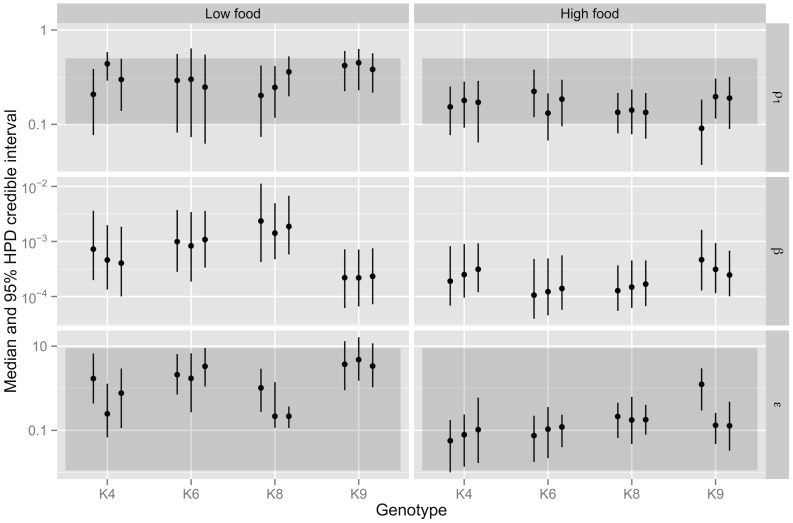
Posterior median and 95%-credible intervals of parameters for the two-wave, *G*-saturating model. The parameters 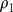, 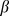 and 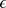 are shown for each population. Darker grey areas show the prior ranges (see Table 1). A vague prior is assigned to 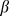.

The models differ considerably in their expectation of the concentration of free infectious parasites (bottom panels in Figure 3; and Figure S2 in File S1): in contrast to the *G*-saturating models, the mass-action and *N*-saturating infection models predict rapid depletion of the inoculum within a few days. Unfortunately this could not be validated with the data available. The huge dip in the inoculum results from high values of the parasite decay rate 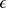, up to an order of magnitude above the prior range (posterior median global estimate for high food treatment: 13.4 with the mass-action model and 11.7 with the *N*-saturating model). The two-wave, *G*-saturating model gives posterior estimates of *ε* compatible with the prior range (Table 3 and Figure 4), providing further support for this model.

**Table 3:** Food-level-specific posterior medians and 95% intervals (Lower, Upper) for overall parameters of the two-wave, *G*-saturating model.

		Low food					High food				
			Global		Genotype			Global		Genotype	
		Median	Lower	Upper	Lower Upper		Median	Lower	Upper	Lower	Upper
*r_S_*	day^−1^	0.22	0.14	0.33	0.059	0.78	0.27	0.18	0.41	0.075	1.01
*k*		3500	2400	5100	1000	12000	8100	5600	12000	2400	27000
*ρ*_1_		0.29	0.17	0.44	0.072	0.67	0.18	0.11	0.30	0.045	0.54
*ρ*_2_		0.64	0.37	0.84	0.20	0.96	0.70	0.47	0.89	0.23	0.98
100*β*	day^−2^	0.068	0.020	0.29	0.0067	0.82	0.021	0.0066	0.068	0.0020	0.21
100*α_G_*		0.20	0.025	1.69	0.0016	26	0.19	0.020	1.3	0.0012	19
*δ*	day^−1^	4.8	1.6	16	0.55	45	14	4.4	47	1.5	130
*m_I_*	day^−1^	0.18	0.086	0.36	0.032	0.96	0.19	0.092	0.37	0.033	0.99
*ε*	day^−1^	1.2	0.35	4.5	0.087	18	0.16	0.050	0.55	0.012	2.4

‘Global’ intervals reflect the degree of uncertainty in estimating *φ_f_*. ‘Genotype’ intervals are *predictive* intervals for the mean parameters (across replicates) of a randomly chosen genotype: they reflect variability between genotypes as well as uncertainty regarding parameter values.

**Figure 5:**
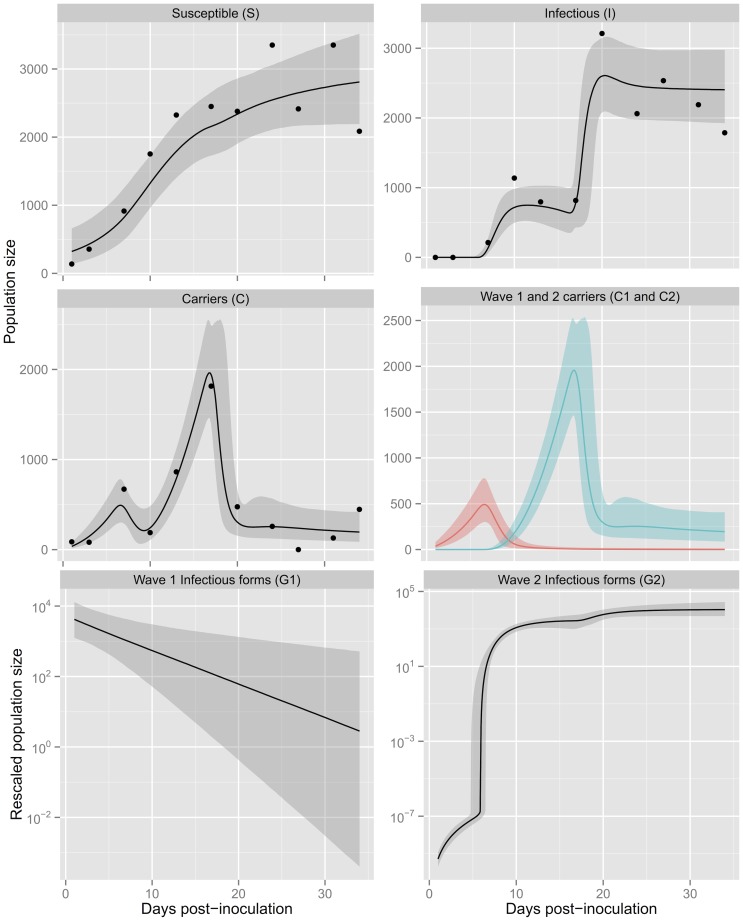
Posterior 95%-credible intervals for clone K8, replicate A, in high food. The dots show experimental data and the lines show the predicted dynamics for the two-wave, 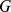-saturating model. In the central panel on the right-hand side, the red line shows the predicted dynamics of 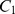 and the blue line the predicted dynamics of 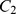.

In the following we thus focus on the two-wave *G*-saturating model because, out of the models we considered, it is the most consistent with the observed time series, both quantitatively (as measured by posterior mean deviance) and qualitatively. As shown in Figure 5 (and Figure S3 in File S1), the Bayesian framework allows us to produce credible intervals for the predicted dynamics: although the model fails to capture some of the fluctuations observed around the end of the experiment, it matches the main observed patterns very well, while highlighting regions with greater uncertainty. In addition, the predicted dynamics of carrier hosts 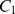 and 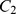 indicate very little overlap between the two waves of infection.

**Figure 6:**
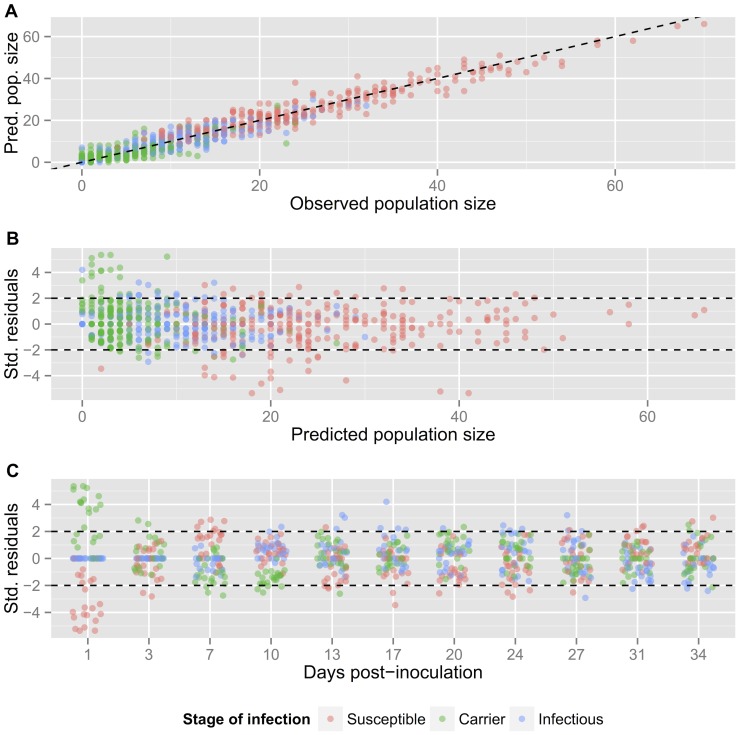
Assessment of model adequacy for the two-wave, 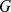-saturating model. (a) Predicted population sizes (posterior medians) against observed population size for each host-type in each replicate. (b) Standardised residuals against predicted population sizes (posterior medians) for each host-type in each replicate. (c) Standardised residuals against number of days post-inoculation. The residuals are jittered horizontally around each observation day to reduce overplotting.

To examine the two-wave 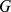-saturating model in more detail we compared the predicted population sizes (posterior medians) against the observations and computed standardised residuals for each of the observed sampled populations. Figure 6A shows predicted vs. observed population sizes and suggests a good overall performance. The standardised residuals are also generally within the expected range (Figure 6B), although some of the susceptible and carrier populations one day post-inoculation have been somewhat overestimated and underestimated, respectively (Figure 6C). Here the model is struggling to account for several early, non-zero carrier populations. As the model-predicted proportion of carriers is very small after one day, the implied sampling variation is also small, and so the standardised residuals are larger than expected. The model also seems to overpredict slightly the number of carriers around days 7–10. We feel that the performance is adequate, however, given the number of data available, which limit the extent to which the model may be extended.

### 3.3 Hierarchical parameter estimation

Our second objective was to estimate the parameters of the model and compare the values across experimental treatments. We analysed separately the dynamics of the infected and uninfected populations. By fitting a logistic growth model (equation (2), with growth rates 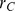 and 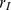 and force of infection 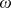 all set to zero) to the time series from the 24 uninfected populations, we obtained posterior distributions for the intrinsic growth rate of uninfected paramecia 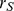, the initial population size 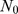 and the population's carrying capacity 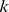. Food level had a clear effect on carrying capacities but no obvious effect on the intrinsic growth rate. Variability between genotypes was apparent at both food levels but more pronounced at low food level. The posterior distributions for 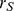 and 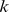 were then used as priors for the parameters in the infected populations, as described in Text S1 in File S1. For the infected populations, we summarise the posterior distributions for the overall parameters of the two-wave, 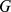-saturating model in Table 3.

The only significant effects for the overall mean parameters over all genotypes were a positive impact of food supply on the carrying capacity 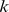 and a negative impact on the parasite's degradation rate 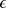. Significant food-level effects were also apparent for some genotypes (but not overall). Figure 4 shows replicate-specific posterior summaries for the threshold parameter 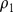, infection rate 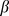 and degradation rate 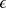. There is the suggestion of a negative food-effect for the infection rate, although this effect is only significant in genotype K8. Additionally, the first-wave threshold for production of infectious parasites 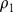 is lower in high food settings, but this effect is only significant in genotype K9. For genotype K9, both the factor controlling saturation of infection 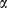 and the additional death rate of infectious hosts 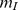 are significantly larger in the high food setting. As explained in section 3.4, the latter effect has implications for the expected persistence of infection.

Experimental measurements on paramecia isolated from the same populations had indicated that hosts carrying infectious forms suffered from reduced survival and replication rates [11]. Posterior estimates of 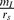 (the death rate as a proportion of the intrinsic growth rate of uninfected paramecia) for the individual populations were typically 79% (mean; interquartile range 64–93%), confirming the high virulence of infectious forms of the parasite. However, we found no significant reduction in division rates, as all posterior credible intervals for 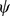 contained 1.

### 3.4 Reproductive ratio

Beyond individual parameter estimates, the Bayesian framework also allows us to estimate composite variables of biological relevance. One aspect of particular interest in infectious disease dynamics is the persistence of the pathogen in a closed host population. In theory, the ability of a pathogen to spread in a population is governed by its basic reproductive ratio (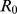), defined as the average number of secondary infections caused by a single infectious agent introduced into a fully-susceptible population of hosts—a value greater than one is necessary for endemic persistence. A common property of many systems is that mortality of hosts, and especially any infection-induced mortality (a trait known as virulence), reduces 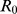.

**Figure 7:**
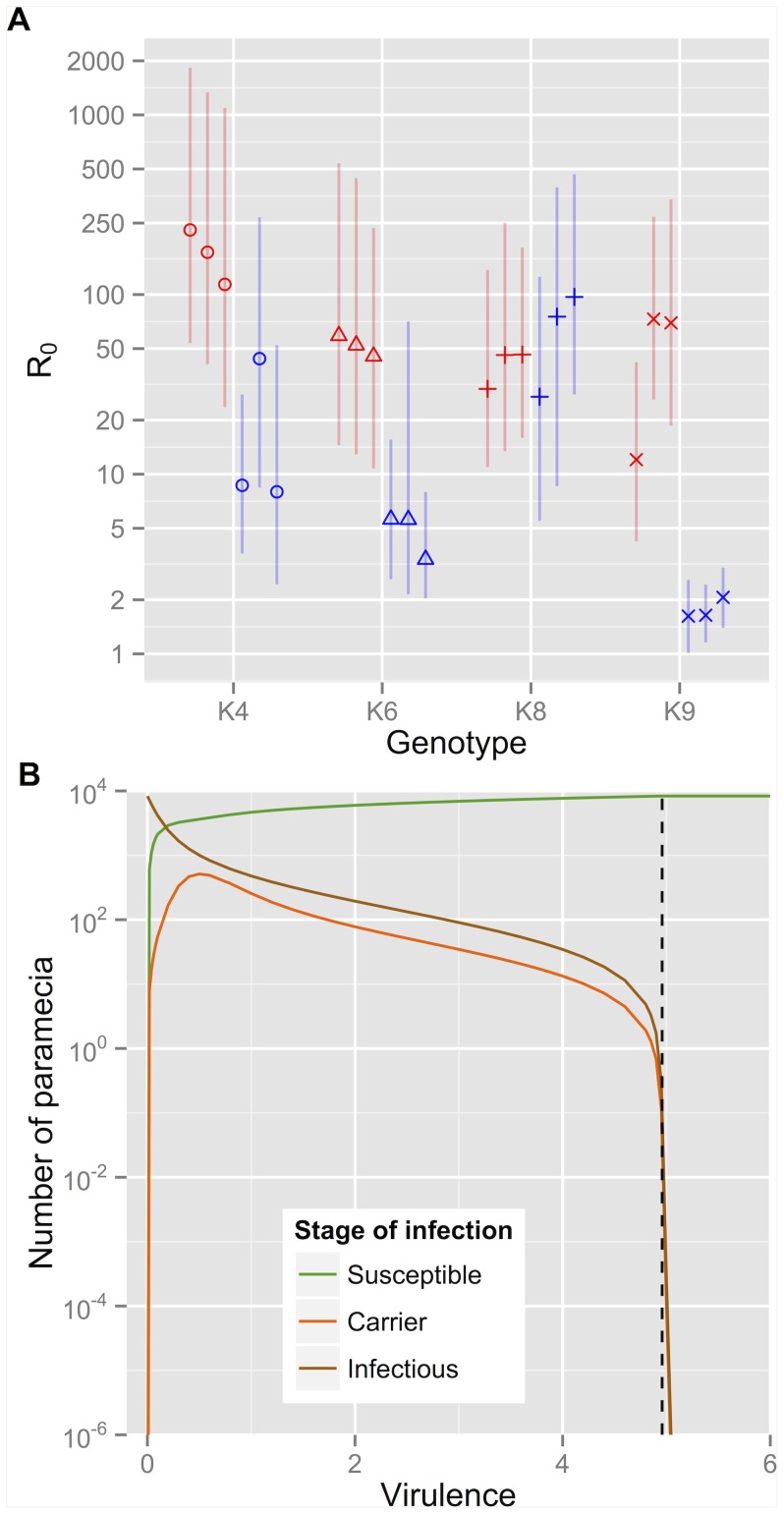
Persistence of infection for the two-wave, 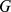-saturating model. (a) Posterior medians and 95%-credible intervals of 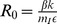 for every experimental population; red: high food, blue: low food. (b) Equilibrium values of 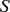, 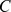 and 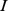 (obtained by running numerical simulations of the model for 5000 days) across a range of values of the virulence 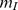, shown here for genotype K8 in high food, replicate A. The vertical dashed line shows the position of the predicted threshold, 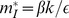, corresponding to 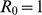.

Our model reduces to a simple horizontal transmission model if we consider the introduction of the pathogen into an uninfected host population at carrying capacity, setting 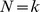 in (2)–(7) and ignoring the first wave of infection. The pathogen's reproductive ratio can be derived from first principles as 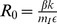 in this scenario. This gives posterior median estimates of 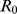 ranging from 12 to 229 in high food populations, and from 1.6 to 97 in low food populations (Figure 7A); except for genotype K8, the estimates of 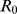 for each genotype are greater in high food than in low food by one order of magnitude (although these differences are only statistically significant for genotype K9). Values for K9 in low food are particularly low, around 2, suggesting that the parasite could be eliminated by, for example, halving the host carrying capacity. By varying each parameter in turn, we verified that infection can spread in model simulations only if the condition 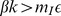 is satisfied. Figure 7B shows one example, highlighting the threshold in virulence 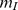.

### 3.5 Dynamics of the two-wave model

**Figure 8:**
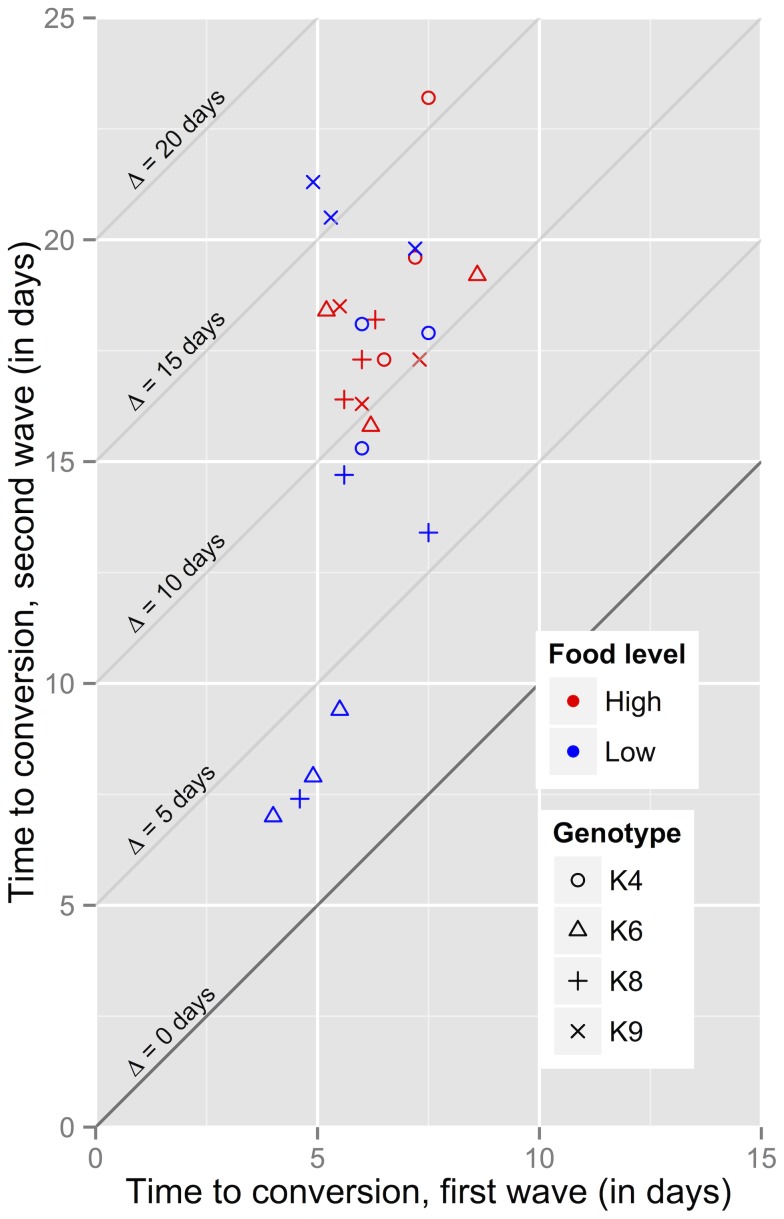
Predicted times to conversion for the two-wave, 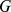-saturating model. The predicted time to conversion from 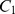 to 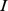 (horizontal axis) and from 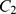 to 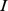 (vertical axis) is shown for every population. These were obtained from numerical solutions the model, using the posterior median parameter values. The diagonal lines represent isoclines of the difference 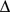 between the delays associated with the first and second waves of infectious form production.

Having selected the two-wave model structure and estimated parameter values consistent with our dataset and prior beliefs, we can use the mechanistic model (2)–(7) to make further predictions about the mechanisms underlying the observed dynamics. Figure 5 clearly shows the two waves of infection generated by the model, based on the densities of carrier hosts 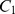 and 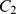. Numerical solutions of the model obtained by using the posterior median parameters for each population give us access to the timing of the two waves. Figure 8 shows the times when infectious forms from the first and second waves start to be produced, i.e. respectively when 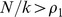 and 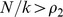 as per (1). In particular, we see that the difference 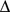 between the delays associated with the first and second waves of infectious form production varies widely from 3 to 17 days in low food treatment, and from 9 to 16 days in high food treatment. This difference 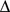 provides us with an estimate of the generation time of infection, complementing the basic reproductive ratio which holds no information on the speed of infection spread.

**Figure 9:**
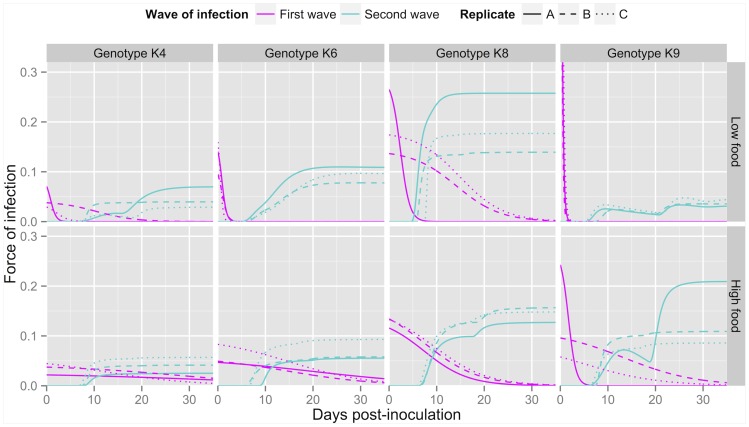
Predicted dynamics of the force of infection for the two-wave, 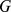-saturating model. These were obtained for every population using the posterior median parameter values. Top row: low food, bottom row: high food. Panels from left to right: genotypes K4, K6, K8 and K9. Magenta: first wave (based on 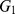), cyan: second wave (based on 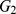).

Finally, we can visualise temporal variations in the force of infection 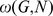, which in the two-wave, 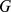-saturating model, is equal to 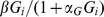 for the 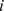-th wave. Figure 9 shows the temporal variations in the forces of infection for both waves, based on the parameters' posterior medians for each of the 24 experimental populations. In most populations, the second wave of infection generates a visible increase in the force of infection around day 20, following the release of newly produced infectious forms. In addition, the fitted models predict that all populations have settled to a steady state by day 34. Interestingly, in most populations the force of infection at steady state is very close to the initial value, with the exception of genotype K9 in low food.

## Discussion

Our study illustrates how a Bayesian statistical framework can be combined with dynamic models to provide extensive analysis of complex longitudinal experimental data. We repeatedly sampled 48 experimental populations of paramecia over 34 days, to assess their size and the proportion of hosts infected with reproductive or infectious forms of the parasite. The populations combined two food levels (high or low), two inoculum types (with or without parasite), four *P. caudatum* genotypes, and each of the resulting 16 treatments was replicated three times. We developed a series of mathematical models that describe different plausible mechanisms for the processes of infection in this system, and aimed to assess their respective abilities to reproduce the experimental data. Instead of fitting models to individual datasets, as is often done when dealing with repeated sampling, we modelled the whole data using a hierarchical statistical model. This approach estimates parameters for each individual population, and also by genotype and food level (Table 3). The ‘borrowing of strength’ effect inherent in hierarchical statistical models means that these estimates efficiently synthesise all information that is available: information on each replicate is augmented by information from other replicates. Although hierarchical Bayesian frameworks have been used to fit dynamic models for the within-host dynamics of infection to longitudinal time-series in experimental [15] and clinical [29] studies, this is one of the first applications to experimental epidemiology.

Our first point concerns the very structure of the infection dynamic model. Gilligan *et al.* [30] combined a logistic growth model and direct transmission to assess the functional form of the transmission rate from a series of experiments on potato plants. However, their simple statistical model (least square minimisation) did not allow comparison of parameter estimates across experimental treatment. This particular issue was one of the motivations for the use of a hierarchical model in the present study. Here, we were able to model explicitly an infection process that included logistic population growth, horizontal infection via free particles, vertical transmission to progeny, and a latency period influenced by population dynamics. The Bayesian framework allowed us to compare alternative hypotheses about two key processes: first, we showed that horizontal transmission is limited by host grazing ability; second, we were able to distinguish the relative contributions of the initial inoculum from parasites released by infected hosts later on. In addition, we used the fitted mechanistic model to infer the range of values of two important epidemiological quantities across experimental treatments: the parasite's basic reproductive ratio and the generation time of infection.

While most host-pathogen models assume transmission by direct contact between infectious and susceptible hosts, we included explicitly the free-living stage of the bacterial parasite. This led us to model infection as a predation process, which is a better representation of the biology of this system. Even though we did not measure the density of free bacteria experimentally, there appeared to be enough information in the data to discriminate between alternative infection models. In line with our understanding of the biology, the model with highest support (out of the models considered) assumes that the rate of infection is limited by the density of hosts, which can be interpreted as a finite rate of grazing by the paramecia. While similar findings are common in studies of predators [31], [32], the implications of grazing functional responses for the dynamics of infection by food-borne pathogens have only recently started to be explored [33], [34].

There remain several open questions about the dynamics of the system. In particular, there was not enough information in the available data to investigate potential effects of host density on the rate of release of infectious parasites. Although we know that this release can take place during host replication and following host death, further experiments would be required to quantify the relative importance of these two routes. In principle, as the population size nears the carrying capacity we might expect that the replication rate would decrease but that the death rate would increase, making it difficult to predict the net effect on parasite production. We hope to be able to use our calibrated model to help with the design of new experiments that could address these questions.

The hierarchical statistical model allowed us to assess variation in parameter values across experimental groups. Our initial motivation was to test the effects of food supply on the population dynamics of a host-pathogen system. Various empirical studies have highlighted two conflicting effects of food availability on host-pathogen dynamics [35]: limited nutrient supply can affect host defences, hence benefitting the parasite; on the other hand, parasites rely on resources from their hosts and may therefore suffer from low food supply. As expected, the strongest effect of food levels in our experiment was a positive impact on the host's carrying capacity. In addition, we found that both the infection rate *β* and the parasite's degradation rate 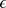 were generally higher in low food treatments (Figure 4). Both effects could be due to higher feeding rates of paramecia on parasites when food is scarce. In order to determine the net effect of food level on the parasite, we determined the posterior distributions of the basic reproductive ratio 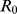 for each population, which revealed a strongly positive effect of food availability on the pathogen's ability to spread in most host genotypes. The only exception was host genotype K8, for which variations in all four parameters that determine 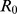 resulted in very similar values for both food levels. Besides, the low values of 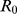 for clone K9 in low food suggest a substantial risk of the parasite failing to establish when introduced in a new host population with a low food concentration. An important caveat is that the basic reproductive rate, which by definition is restricted to the first cycle of infection, takes only horizontal transmission into account. Vertical transmission is an essential feature of this particular parasite, enabling it to persist at a high prevalence.

In conclusion, this study demonstrates how mechanistic models can be fitted to a multi-factorial experimental dataset, using a hierarchical Bayesian framework, to make inference on infection dynamics. A wide range of experimental systems could benefit from this approach, which allows the integration of information from other experiments into prior parameter distributions, the generation of posterior parameter distributions within and across experimental treatments, and the comparison of multiple mechanistic models allowing predictions on fundamental biological processes. The freely available WinBUGS software, which can be easily combined with the widely used R program, provides a user-friendly interface for the implementation of the whole framework.

## Supporting Information

File S1 **Supporting descriptions of how prior distributions were obtained (Text S1), further discussion of identifiability issues (Text S2), as well as supporting Figures S1–S3.**
(PDF)
