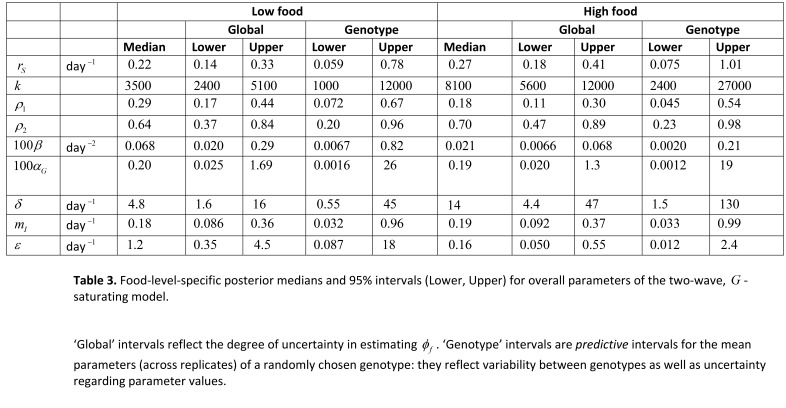# Correction: Modelling the Dynamics of an Experimental Host-Pathogen Microcosm within a Hierarchical Bayesian Framework

**DOI:** 10.1371/annotation/54272e46-d8d3-4845-b630-7ea515b1d652

**Published:** 2013-09-30

**Authors:** David Lunn, Robert J. B. Goudie, Chen Wei, Oliver Kaltz, Olivier Restif

As a result of errors in the production process, Tables 1 and 3 have not been formatted properly.

In Table 1, two rows should have been merged. The correct table can be viewed here: 

**Figure:**
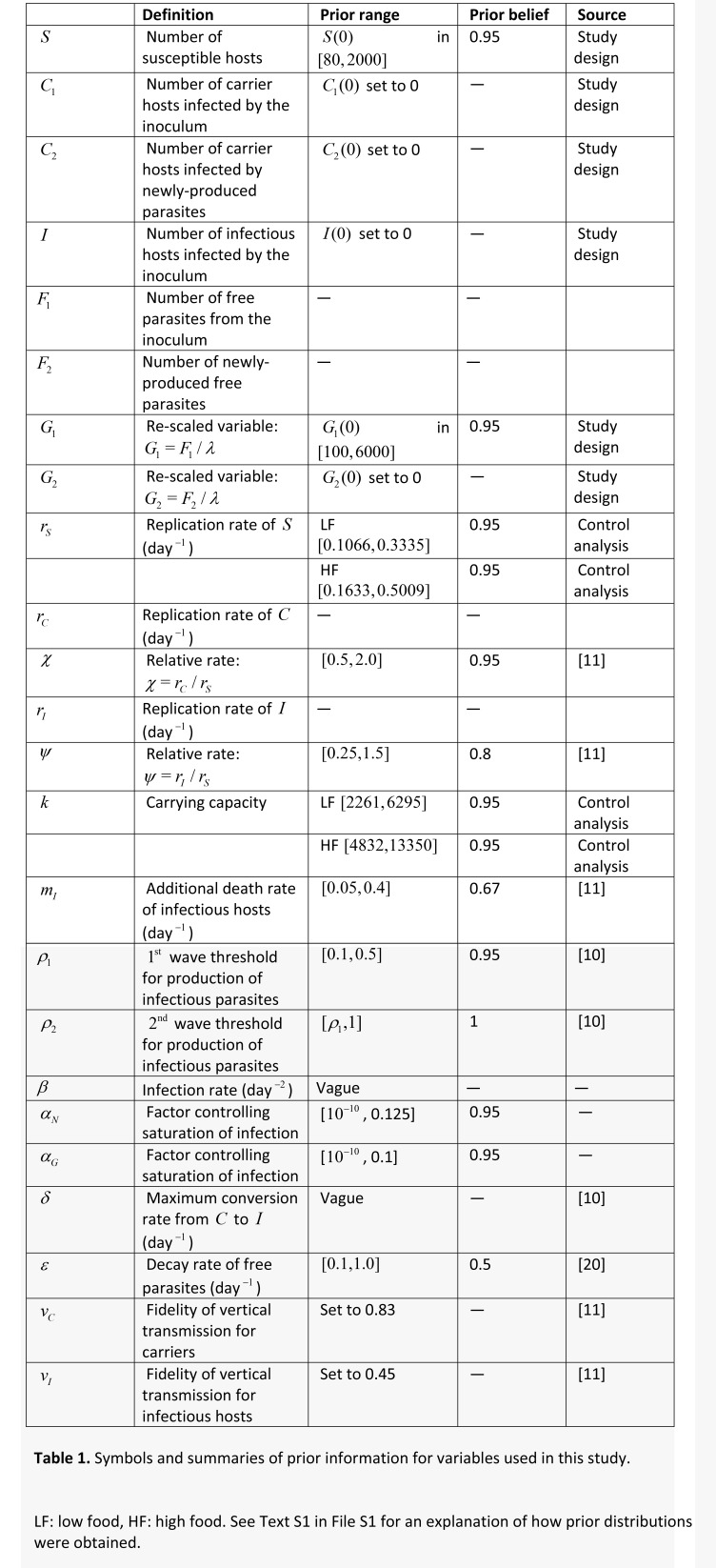


In Table 3, the headers in columns 6 and 7 were mistakenly merged. The correct table can be found here: 

**Figure:**