# Supplementary material for: Modelling the Dynamics of an Experimental Host-Pathogen Microcosm within a Hierarchical Bayesian Framework
Source: PLoS One. 2013 Aug 2;8(8):e69775. doi: 10.1371/journal.pone.0069775 (PMC3732293; doi:10.1371/journal.pone.0069775)
Supplement: File S1 — Supporting descriptions of how prior distributions were obtained (Text S1), further discussion of identifiability issues (Text S2), as well as supporting Figures S1–S3. (PDF) [file pone.0069775.s001.pdf]

File S1: Supporting Information for "Modelling the dynamics of an experimental host-pathogen microcosm within a hierarchical Bayesian framework"

David Lunn[a], Robert J. B. Goudie[a,†], Chen Wei[a,†], Oliver Kaltz[b], and Olivier Restif[c,*]

[a]*MRC Biostatistics Unit, Institute of Public Health, Cambridge, UK*
[b]*Institut des Sciences de l'Evolution, CNRS UMR 5554, Université Montpellier 2, 34095 Montpellier Cedex 05, France*
[c]*Disease Dynamics Unit, Department of Veterinary Medicine, University of Cambridge, Cambridge, UK*
†*Contributed equally*
**E-mail: Corresponding or226@cam.ac.uk*

Text S1: Prior distributions

Our statistical model is completed by the specification of prior distributions for the initial conditions $X^{(S)}_{fgi0}$ and $X^{(G_1)}_{fgi0}$, and the parameters σ_1, σ_2, ϕ_1, ϕ_2, Σ_θ and Σ_μ. We first consider the general distributional form of the priors that we assign to each parameter, and then describe the specific distributional-parameter values.

1.1 General distributional form assumptions

We express prior uncertainty about ϕ_1, ϕ_2, Σ_θ and Σ_μ through the following distributions (with known parameters):

$$\begin{aligned} \phi_f &\sim \text{MVN}_{11}(q_f, Q_f), \qquad f = 1, 2, \\ \Sigma_\theta^{-1} &\sim \text{Wishart}(d_\theta, d_\theta D_\theta), \\ \Sigma_\mu^{-1} &\sim \text{Wishart}(d_\mu, d_\mu D_\mu), \end{aligned}$$

where q_1, Q_1, q_2 and Q_2 are chosen to reflect the extent of prior knowledge regarding each parameter in the mechanistic model, as described below.

We also require prior distributions for the initial conditions $X^{(S)}_{fgi0}$, $X^{(G_1)}_{fgi0}$ and the variance parameter σ_f. We assign a log-normal prior to each of these quantities, which reflects the constraint that each must be positive.

1.2 Specific distributional-parameter values

Recall that ϕ_f, $f = 1, 2$, contains the global mean values of $\{\text{logit}(\rho_1, \nu), \log(\beta, S_{G/N}, k, \delta, m_I, \epsilon, r_S, \chi, \psi)\}$. Thus to set the prior for each component of ϕ_f, we apply the appropriate transformation to the prior information detailed in Table 1.

Most of this prior information is sourced from previous studies (1; 2; 3), but for some parameters no previous study provided suitable information. For this reason we used vague priors for the infection rate β and the maximum conversion rate δ, while for the replication rate r_S and the carrying capacity k we constructed priors from analysis of the control populations (mock inoculum), for which the mechanistic model is given by equation 2 with r_C, r_I and ω all set to zero. The statistical model is as described above but with only 2 parameters instead of 11, and with priors for σ_1, σ_2, ϕ_1, ϕ_2, Σ_θ and Σ_μ all vague.

We chose our prior for $\alpha_{G/N}$ by assuming that at the outset of the experiment the infection process is not fully saturated, as suggested by earlier work (2). This enabled us to derive an upper bound on the saturation factors α_G and α_N, via the direct implication that $\alpha_G G(t)$ and $\alpha_N S(t)$ are not too large at $t = 0$. In the former case we assume that $\alpha_G G(0) < 10$ and, defining $G(0)_{\text{min}}$ as the lower bound of our 95% prior range of belief for $G(0)$, we conclude that $\alpha_G < 10 \times G(0)^{-1}_{\text{min}} = 0.1$. Similarly, we assume $\alpha_N < 10 \times S(0)^{-1}_{\text{min}} = 0.125$. We take these values as 95% prior upper bounds, with a corresponding lower bound of 10^{-10} for both parameters.

The matrices D_θ and D_μ represent our best prior guesses at the values of the corresponding covariances. We set the off-diagonal elements of each matrix to zero, and the diagonal elements corresponding to r_S and k to their respective posterior medians from the control analysis. Thus, the corresponding elements are respectively set in D_θ to 0.1093 and 0.07258, and in D_μ to 0.2068 and 0.1820. In the absence of any prior information on the between-replicate variation of other parameters we use the average of the variabilities of r_S and k from the control analysis. The average is taken on the standard deviation scale, giving $0.0900 = \{(\sqrt{0.1093} + \sqrt{0.07258})/2\}^2$ for all diagonal elements of D_θ relating to the remaining parameters. Similarly, we do not have any prior information on the between-genotype variation of the parameters apart from r_S and k, so similarly we use the variability of these two parameters as a guide, specifying $0.1942 = \{(\sqrt{0.2068} + \sqrt{0.1820})/2\}^2$ for the remaining diagonal elements of D_μ. We wish to remain vague about Σ_θ and Σ_μ, however, and so we set $d_\theta = d_\mu = 11$ to make their priors as uninformative as possible.

For the initial conditions $X^{(S)}_{fgi0}$ and $X^{(G_1)}_{fgi0}$ we assign (on the log scale) prior mean 5.991 and 6.652, and standard deviation 0.8211 and 1.044 respectively. This reflects our prior belief that the initial number of hosts and scaled (by λ^{-1}) number of parasites is unlikely (probability 0.05) to fall outside the ranges $[80, 2000]$ and $[100, 6000]$, respectively.

Finally, we assign (on the log scale) a prior mean 0 and standard deviation 100 for σ_f, reflecting prior ignorance.

Text S2: Identifiability issues

We found when fitting a full hierarchical model for θ that the parameter estimates for the relative rates of reproduction χ and ψ were widely divergent from our prior beliefs. This is a consequence of the non-linearities and the large number of parameters in our model, which can result in the fitting algorithm producing extreme parameter estimates in an attempt to reconcile fully model and data. It is possible that extreme parameter values may highlight the possibility of a deficient model, but, within the context of the chosen model, inferences are only meaningful if

parameters have plausible values. Hence we endeavoured to ensure that all parameter estimates are consistent with the prior beliefs expressed in Table 1.

This proved very difficult with a fully hierarchical model. The prior beliefs expressed in Table 1 are applied to the *overall* parameters in our model, ϕ_f, $f = 1, 2$. Genotype-level and replicate-level parameters are free to go beyond the scope of those priors, however, within the confines of the between-genotype covariance Σ_μ and the within-genotype covariance Σ_θ. With vague priors on the covariance terms, some of the genotype- and replicate-level parameters (χ and ψ) become implausible, because there is very little information in the data regarding the covariances (due to the small numbers of genotypes and replicates within genotypes), and so estimation of the parameters is driven almost entirely by the replicate-level data.

One way of restricting the parameters' freedom is to specify informative priors for Σ_μ and Σ_θ that correspond to small variances. This works to an extent with our data but the priors required to rein in the genotype- and replicate-level parameters to plausible values are implausible themselves, not to mention arbitrarily defined.

We adopted a pragmatic solution to this problem of applying a simplistic, non-linear model to a limited data set: for the problematic quantities, χ and ψ, we applied prior distributions directly to the replicate-level parameters. Hence they are not assumed drawn from some within-genotype 'population' distribution, and their estimates are constrained to be plausible. The price paid to achieve this is that we cannot make inferences about the between- and within-genotype variability of these parameters, though we can about the other parameters.

References

1. Kaltz O, Koella JC (2003) Host growth conditions regulate the plasticity of horizontal and vertical transmission in *Holospora undulata*, a bacterial parasite of the protozoan *Paramecium caudatum*. Evolution 57: 1489-1497.

2. Fels D, Vignon M, Kaltz O (2008) Ecological and genetic determinants of multiple infection and aggregation in a microbial host-parasite system. Parasitology 135: 1373-1383.

3. Restif O, Kaltz O (2006) Condition-dependent virulence in a horizontally and vertically transmitted bacterial parasite. Oikos 114: 148-158.

Supporting figures

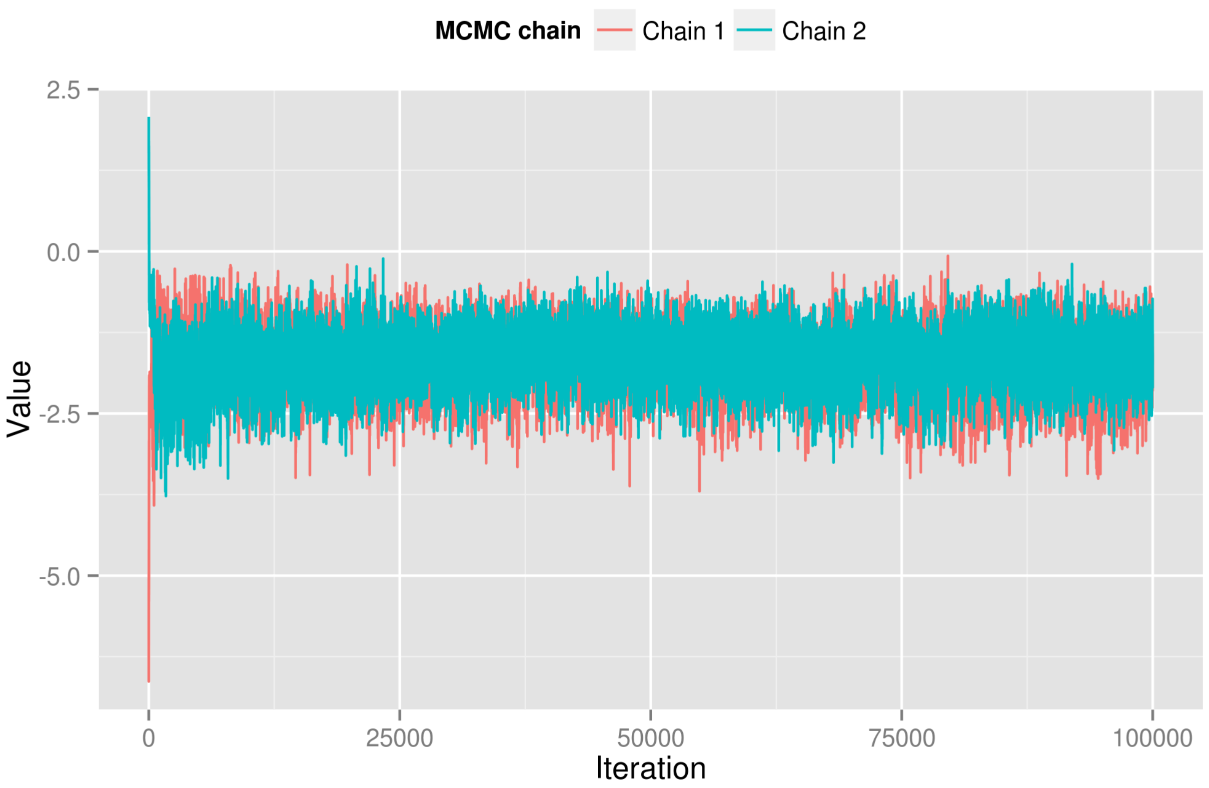

Figure S1. Illustrative chain-history plot Convergence was visually assessed by examining the chain-history plots from two Markov chains, starting from widely differing initial values. The chain-history plot for first component of μ_{11} is shown as an illustrative example.

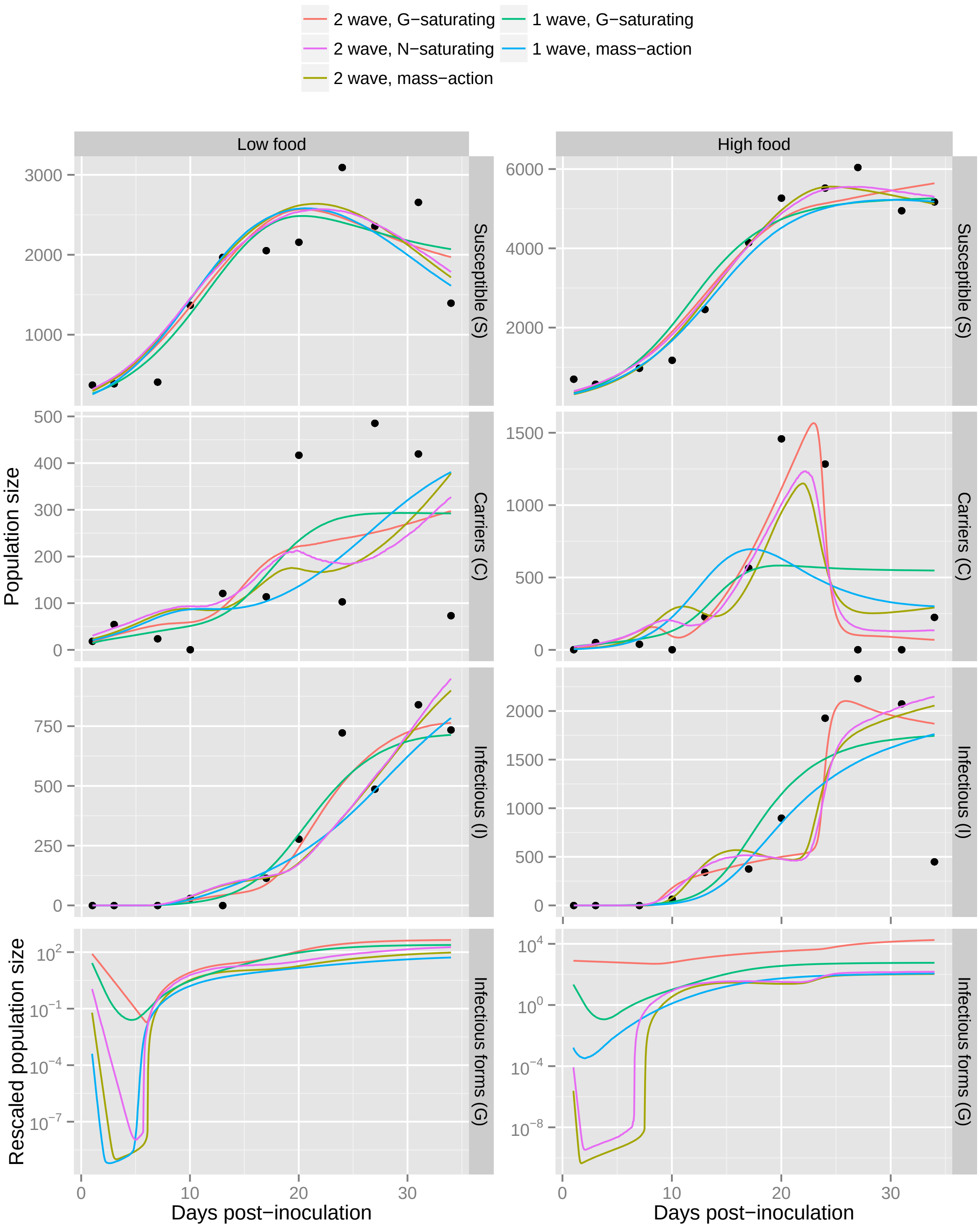

(a) Model fit for clone K4, replicate A

Figure S2. Model fits for the clone and replicate shown in the sub-figure caption in Low food (left) and High food (right). Each panel shows a different variable (from top to bottom: S, C, I and G). The dots show experimental data and the lines the predicted dynamics obtained from each of the five fitted models.

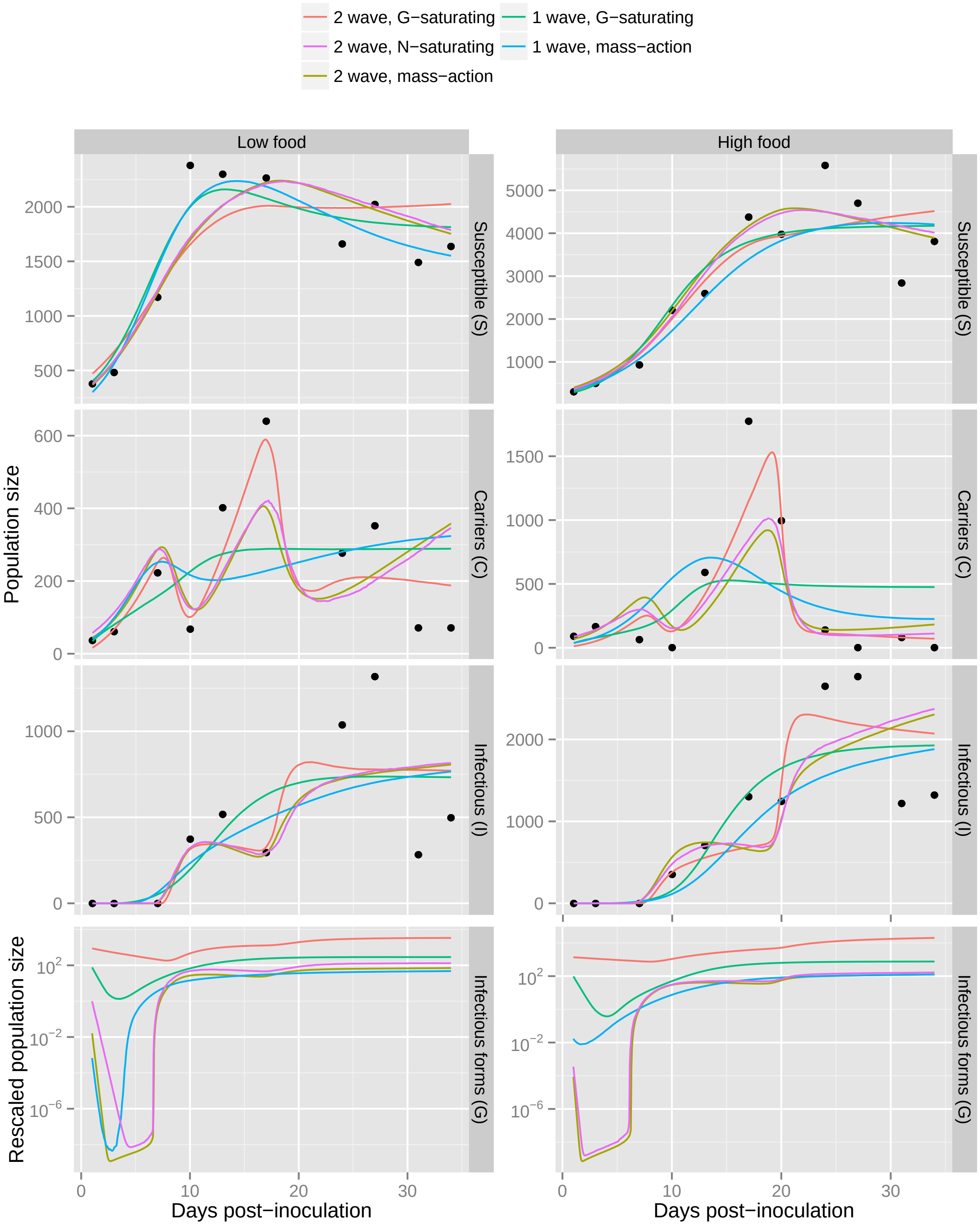

(b) Model fit for clone K4, replicate B

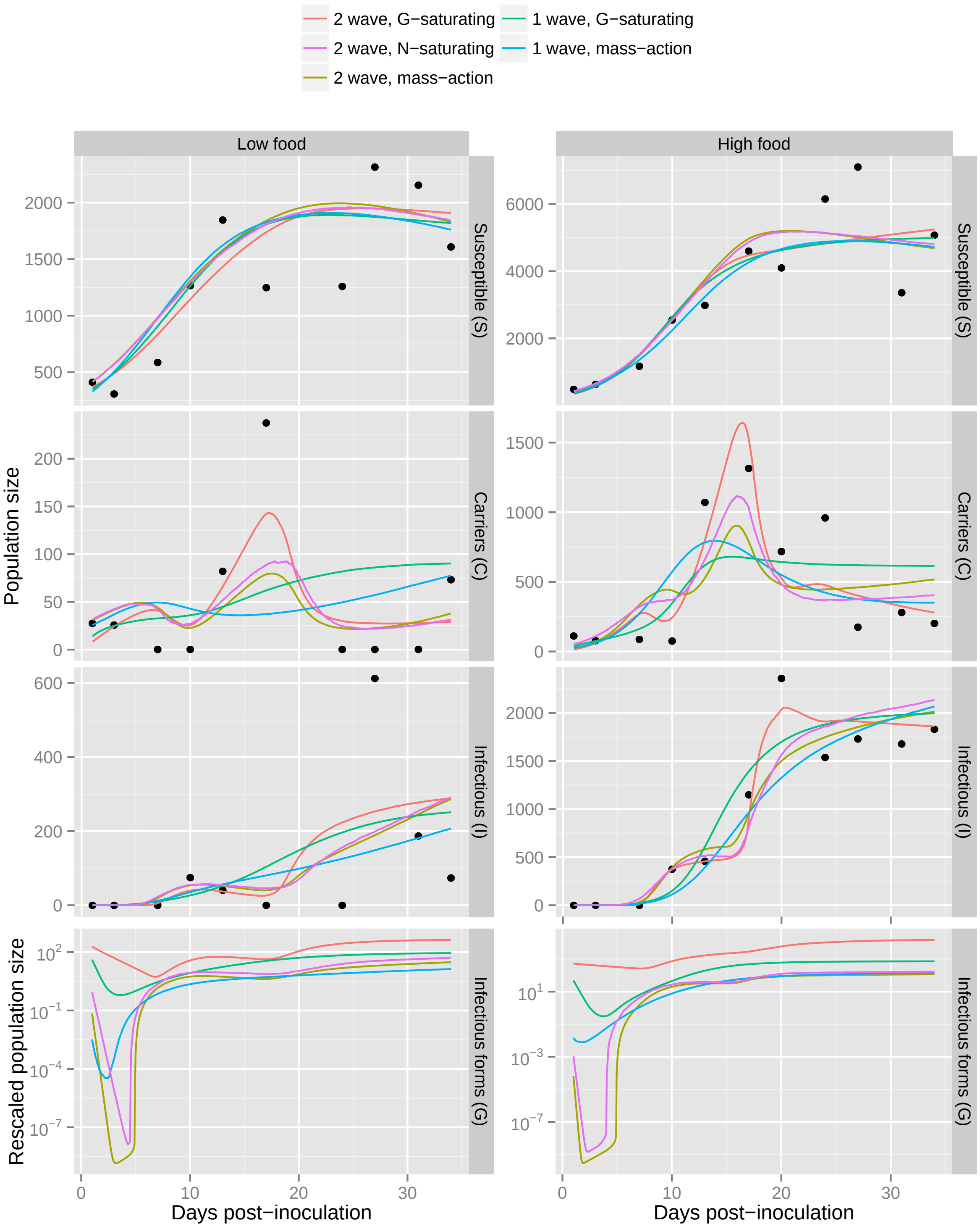

(c) Model fit for clone K4, replicate C

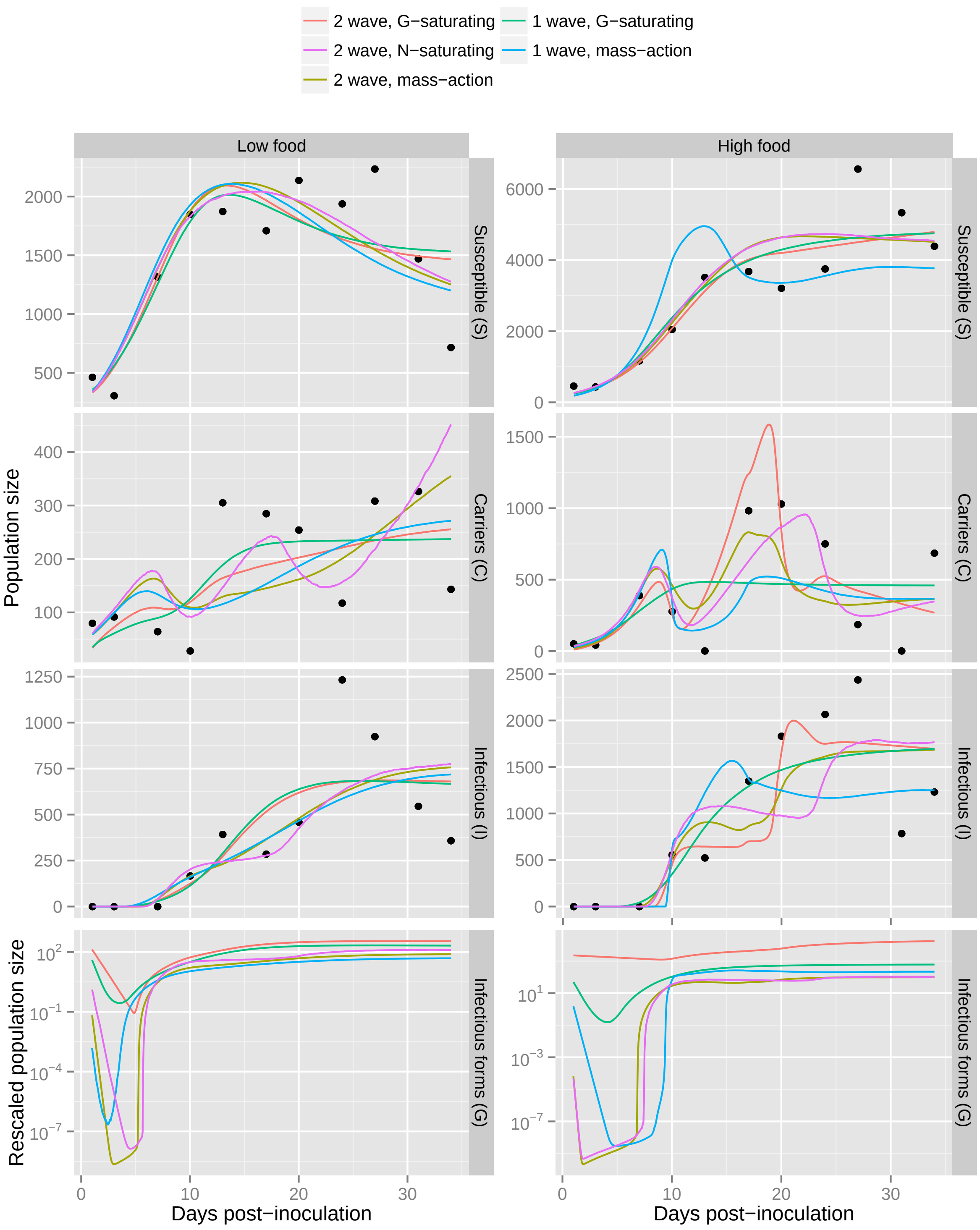

(d) Model fit for clone K6, replicate A

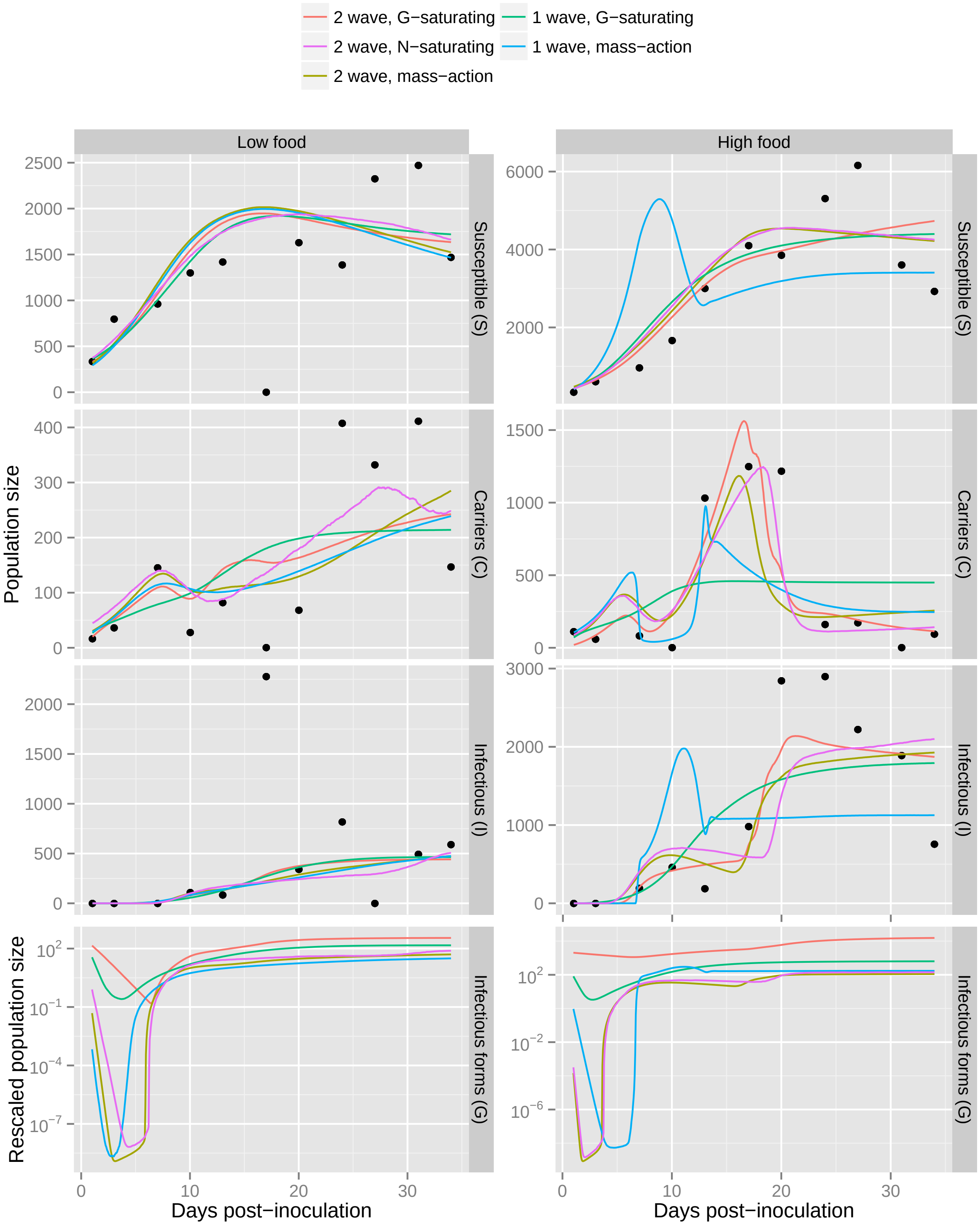

(e) Model fit for clone K6, replicate B

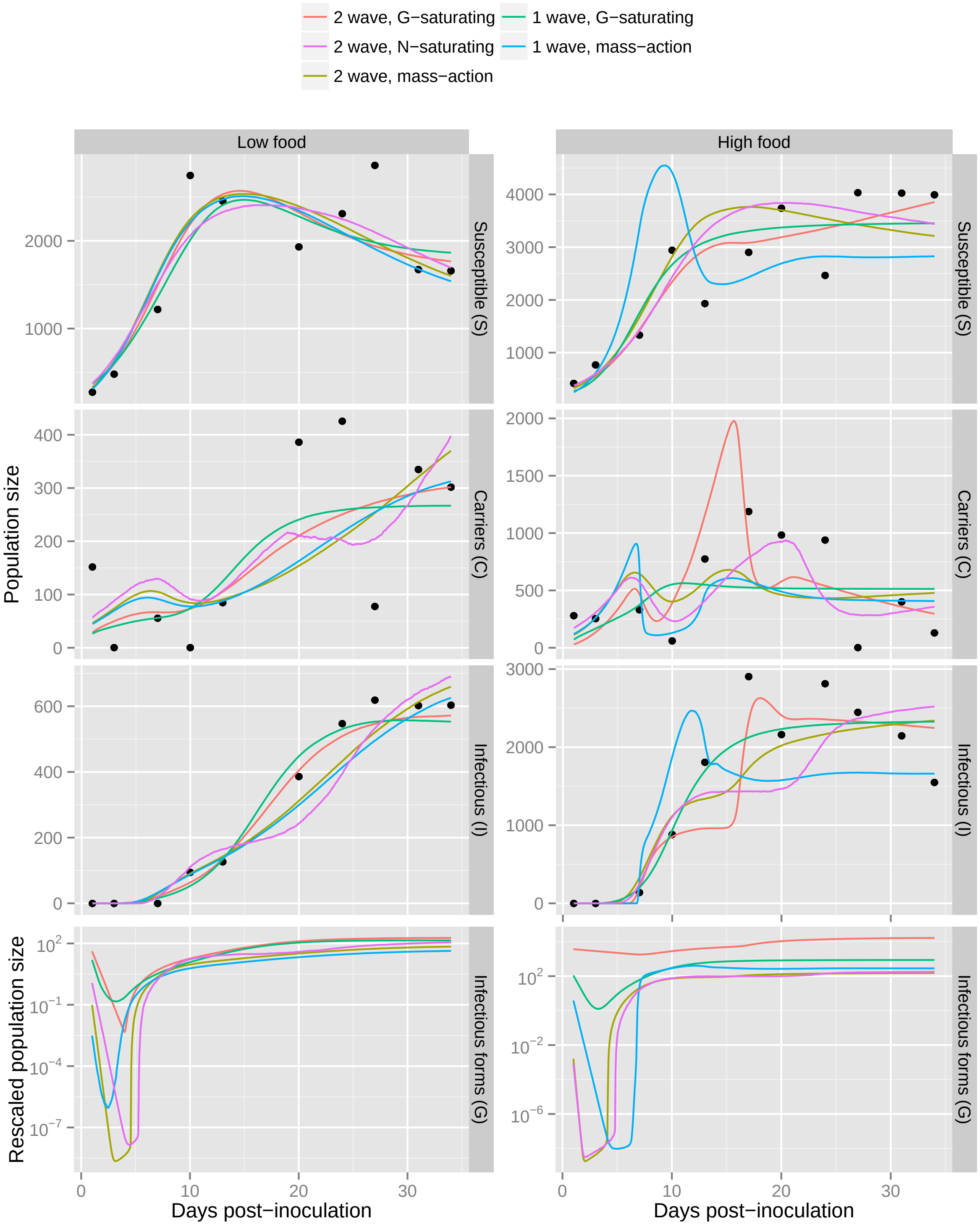

(f) Model fit for clone K6, replicate C

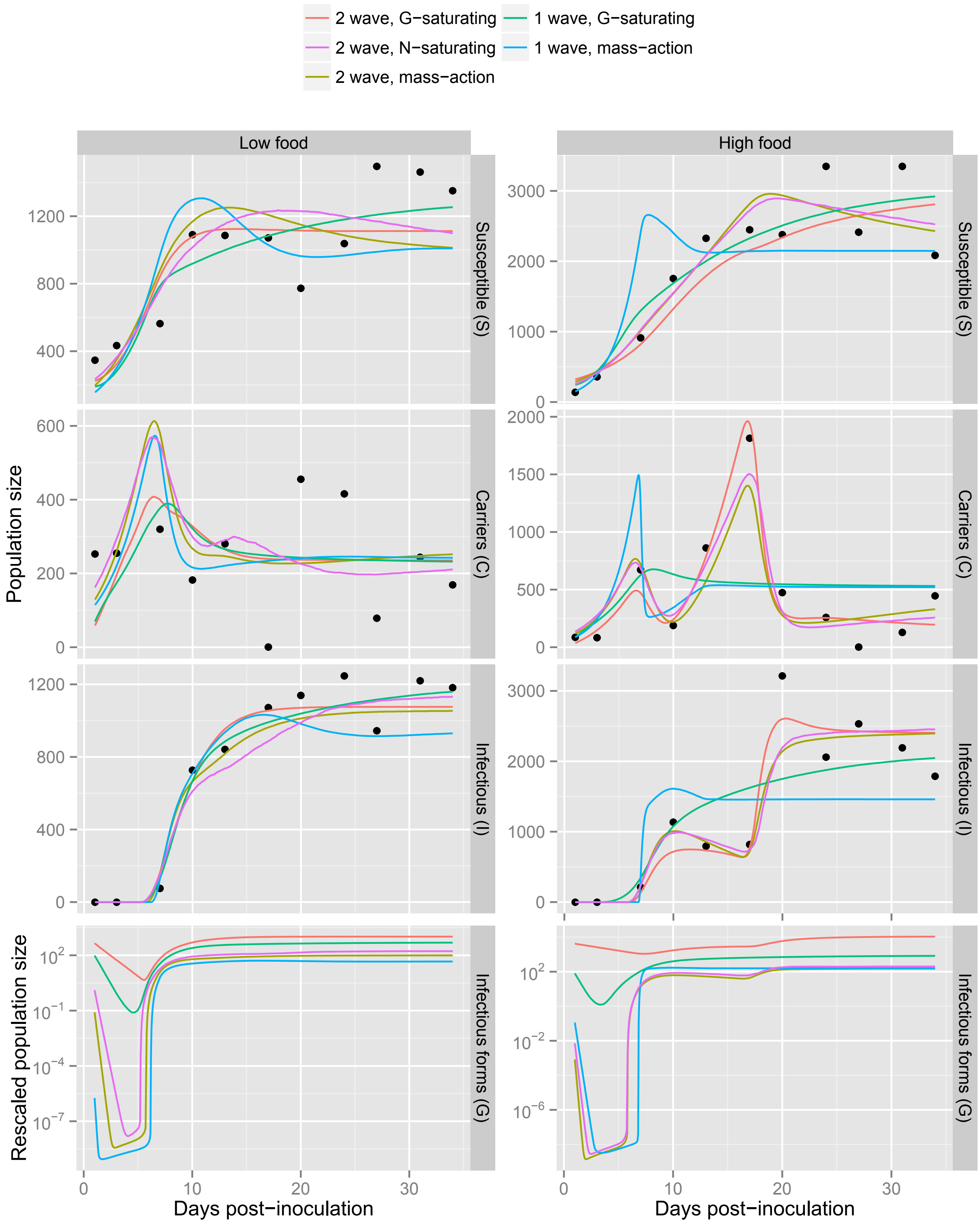

(g) Model fit for clone K8, replicate A

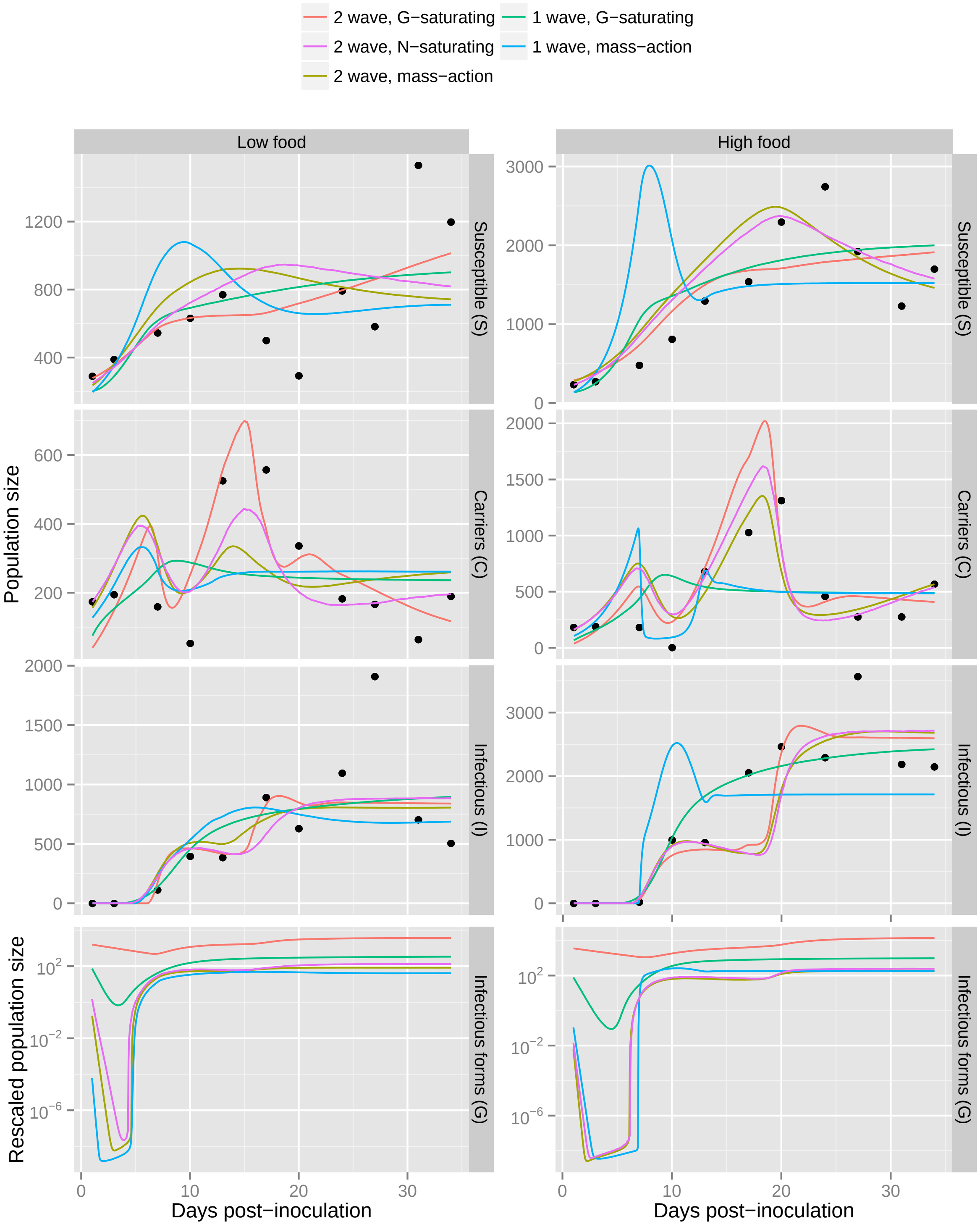

(h) Model fit for clone K8, replicate B

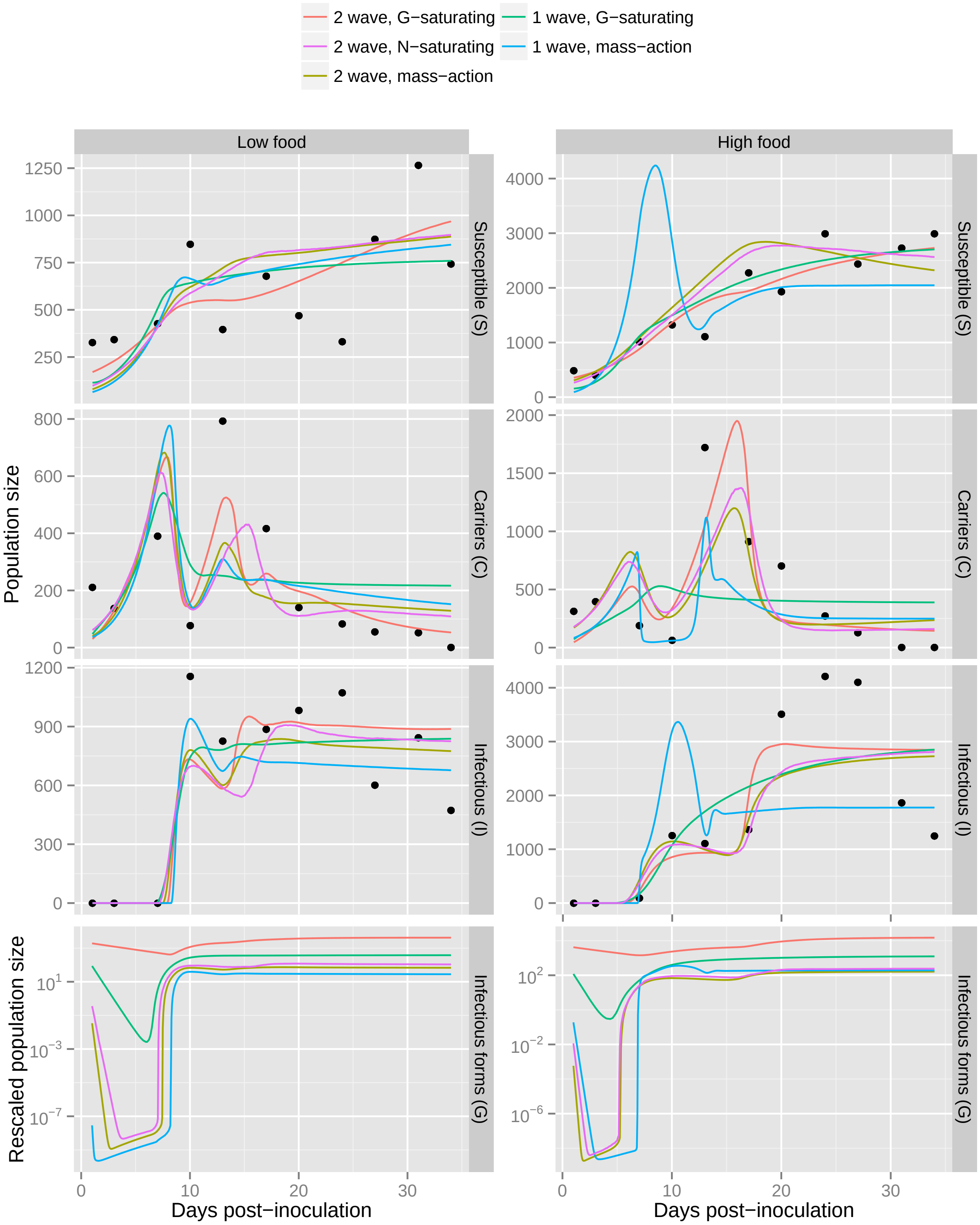

(i) Model fit for clone K8, replicate C

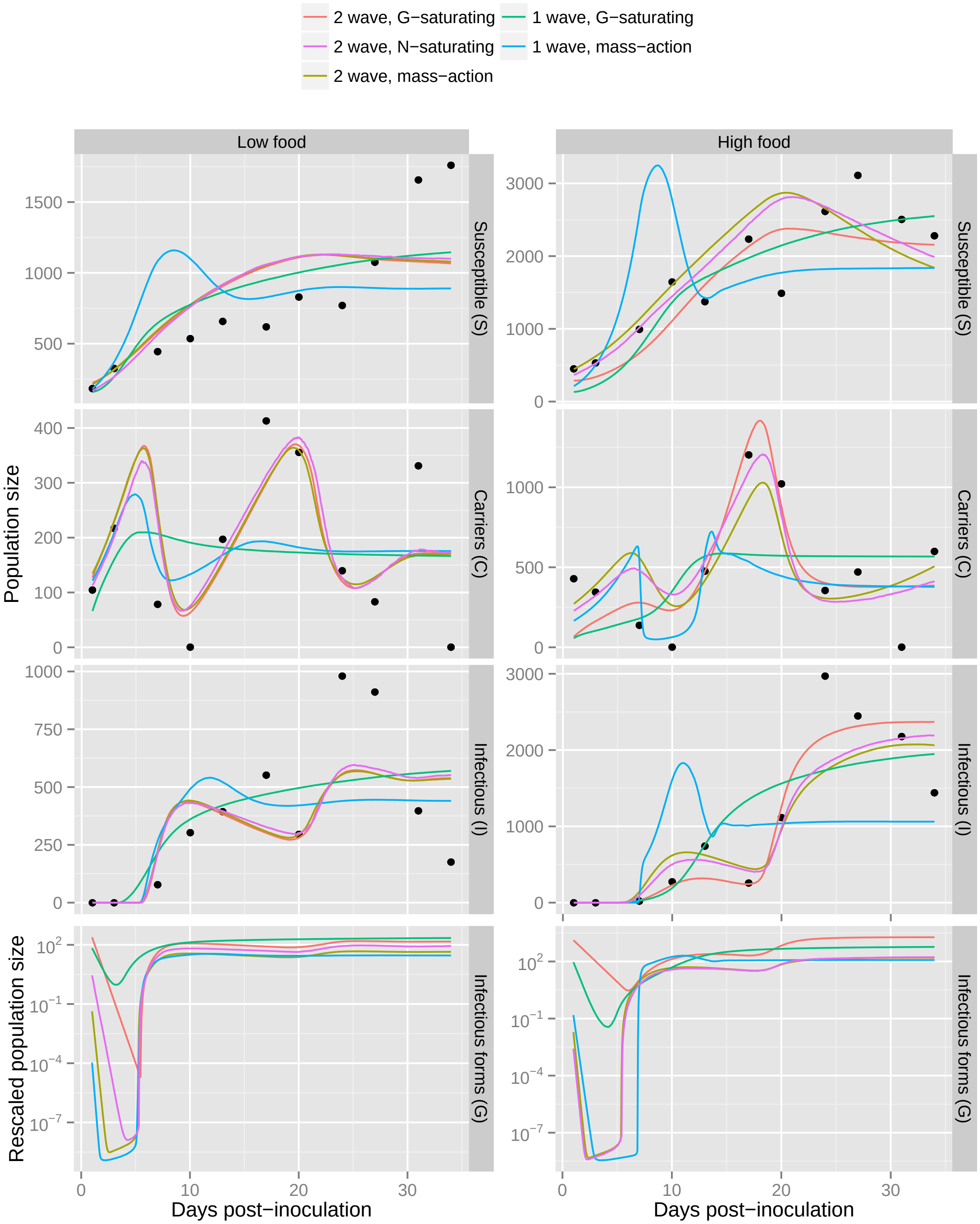

(j) Model fit for clone K9, replicate A

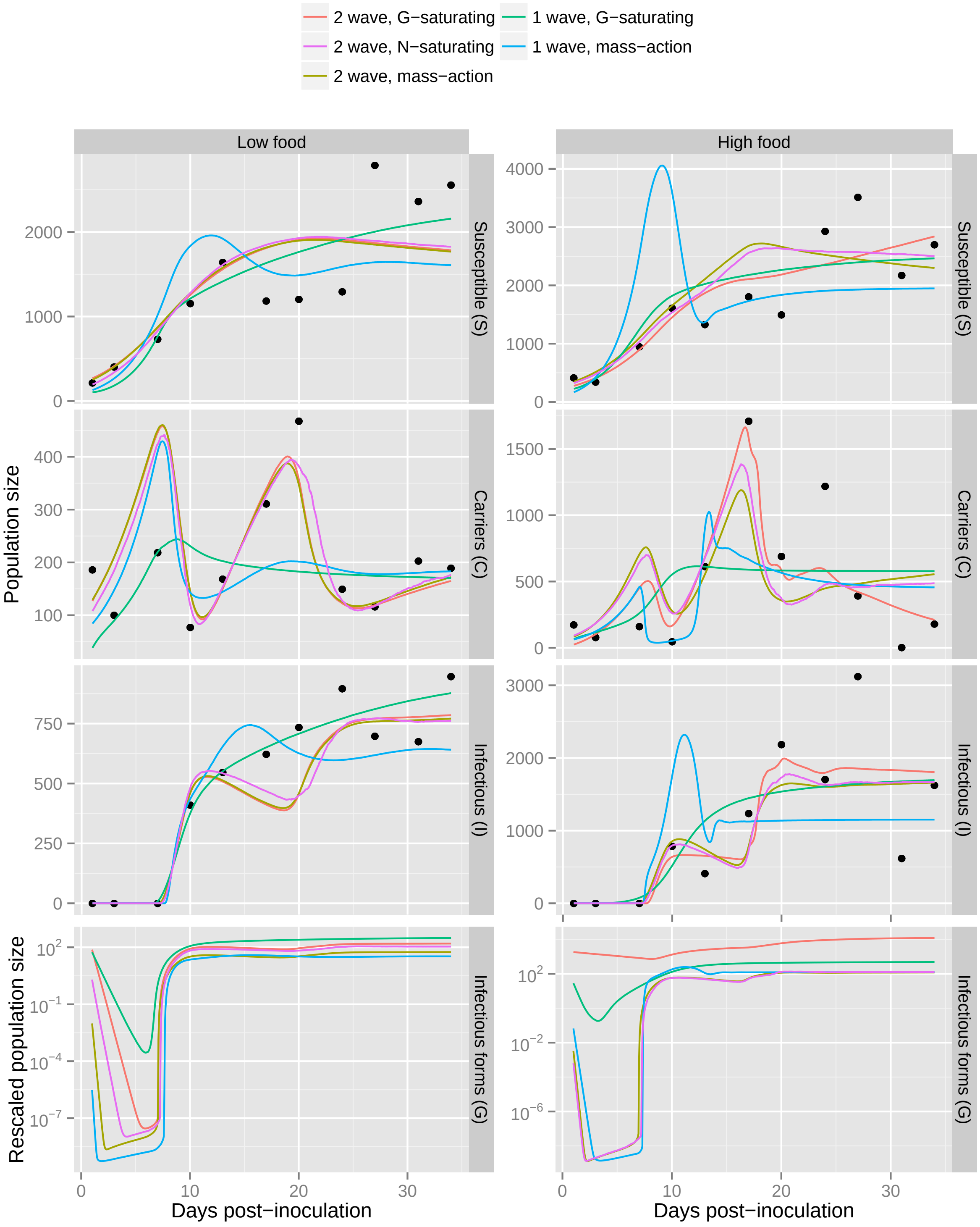

(k) Model fit for clone K9, replicate B

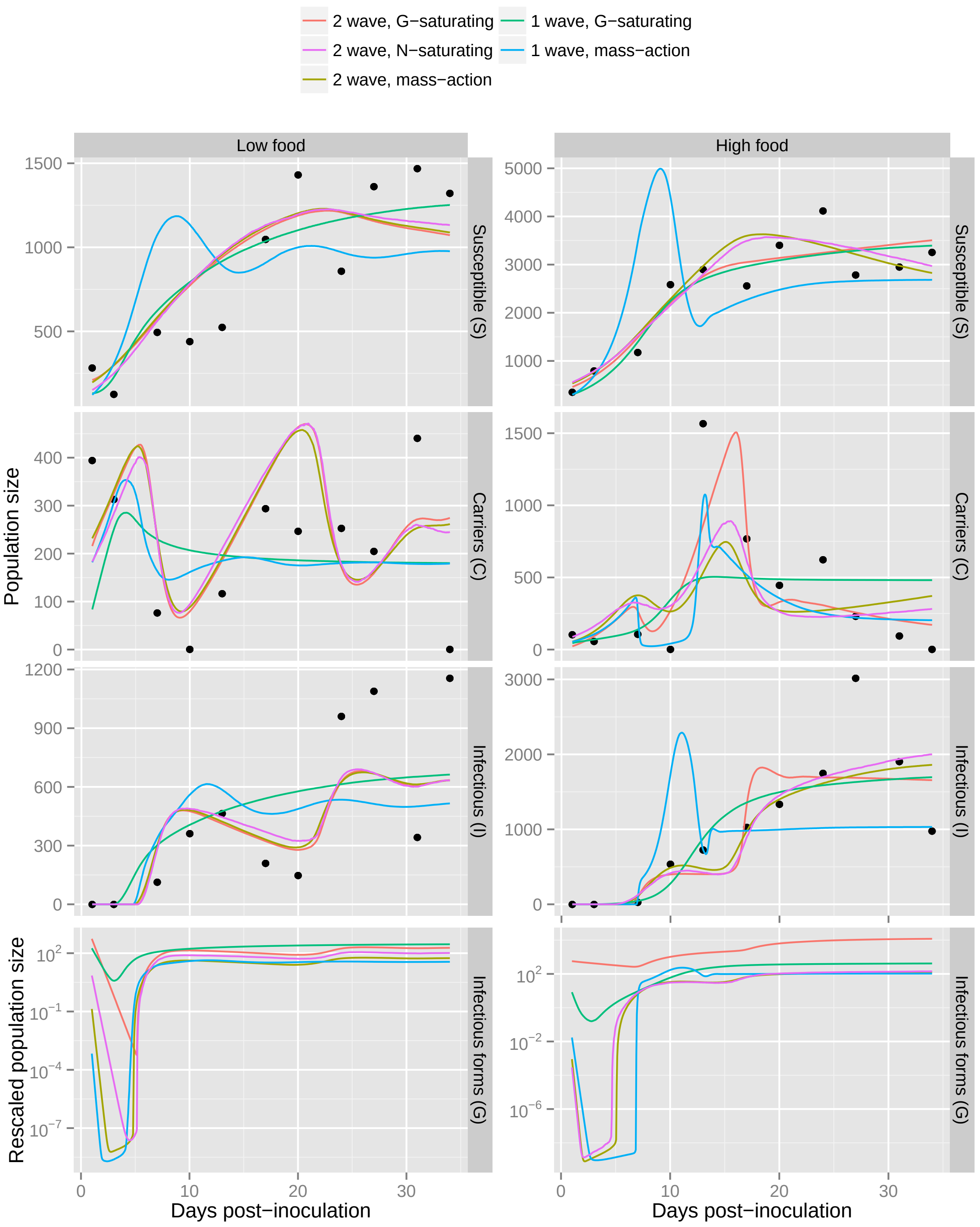

(I) Model fit for clone K9, replicate C

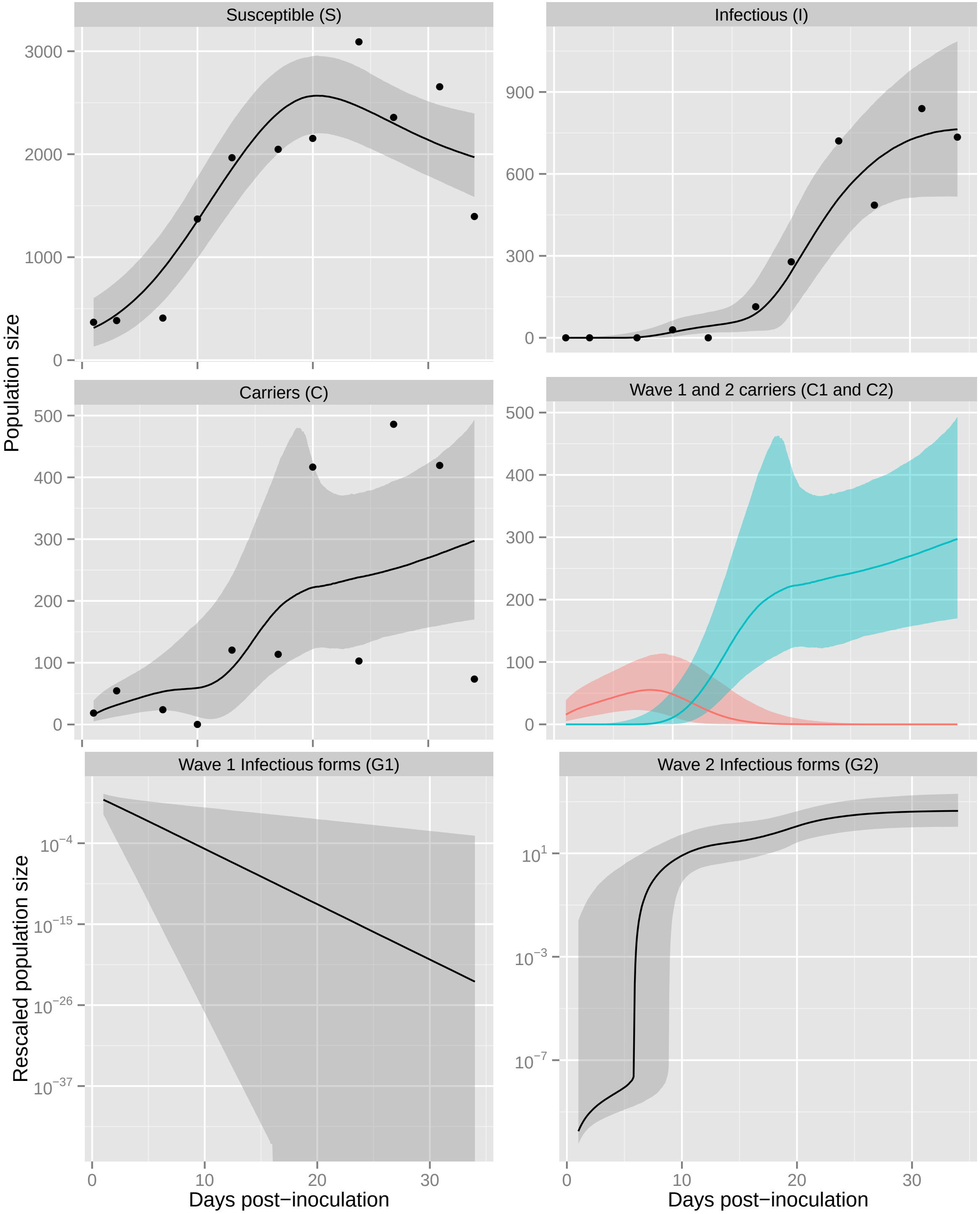

(a) Model fit for clone K4, replicate A, Low food

Figure S3. Best model (two-wave, G-saturating) fitted to the clone, replicate and food level shown in the sub-figure caption, with 95% credible intervals. The dots show experimental data and the lines the predicted dynamics. In the central panel on the right-hand side, the red line shows the predicted dynamics of C_1 and the blue line the predicted dynamics of C_2.

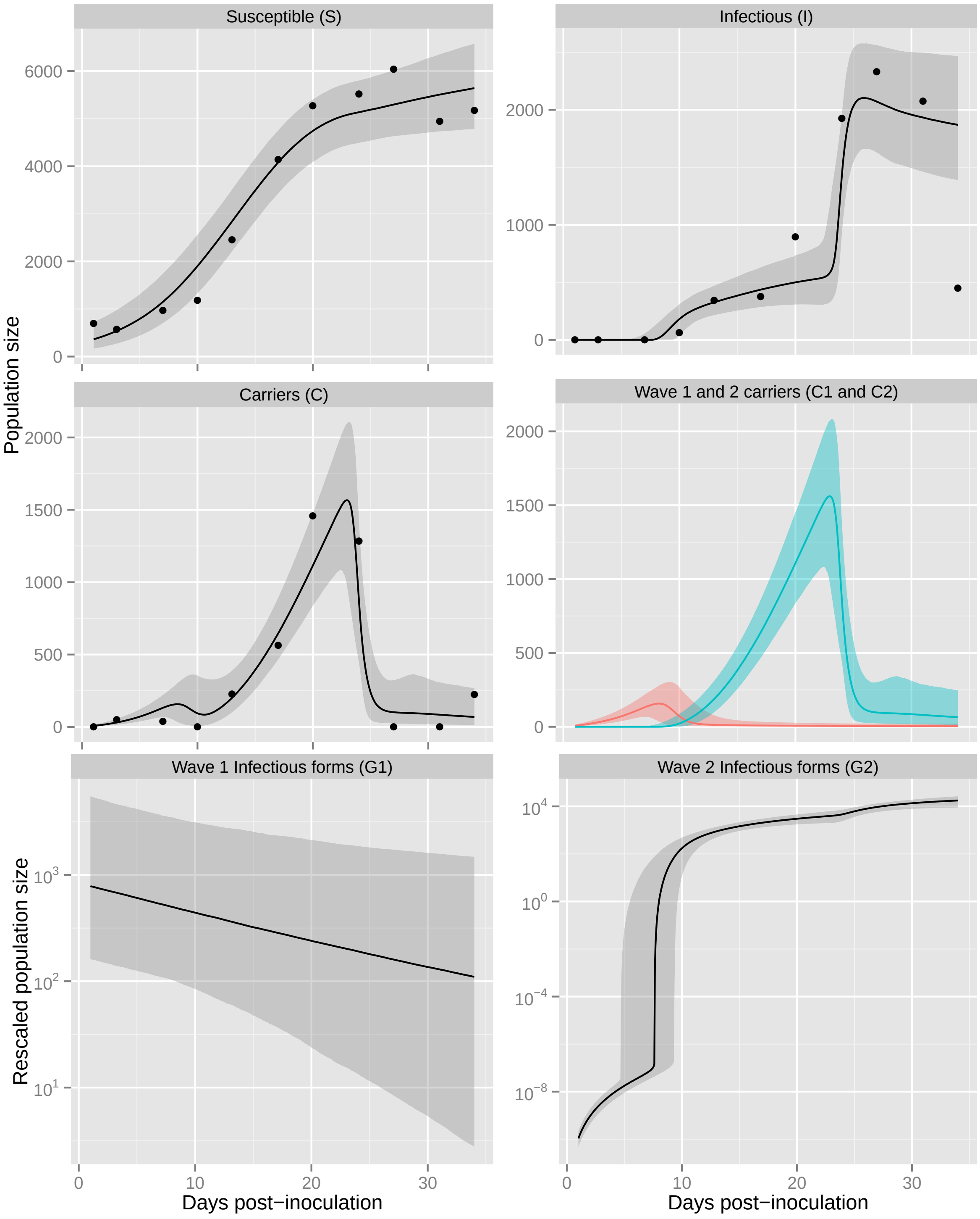

(b) Model fit for clone K4, replicate A, High food

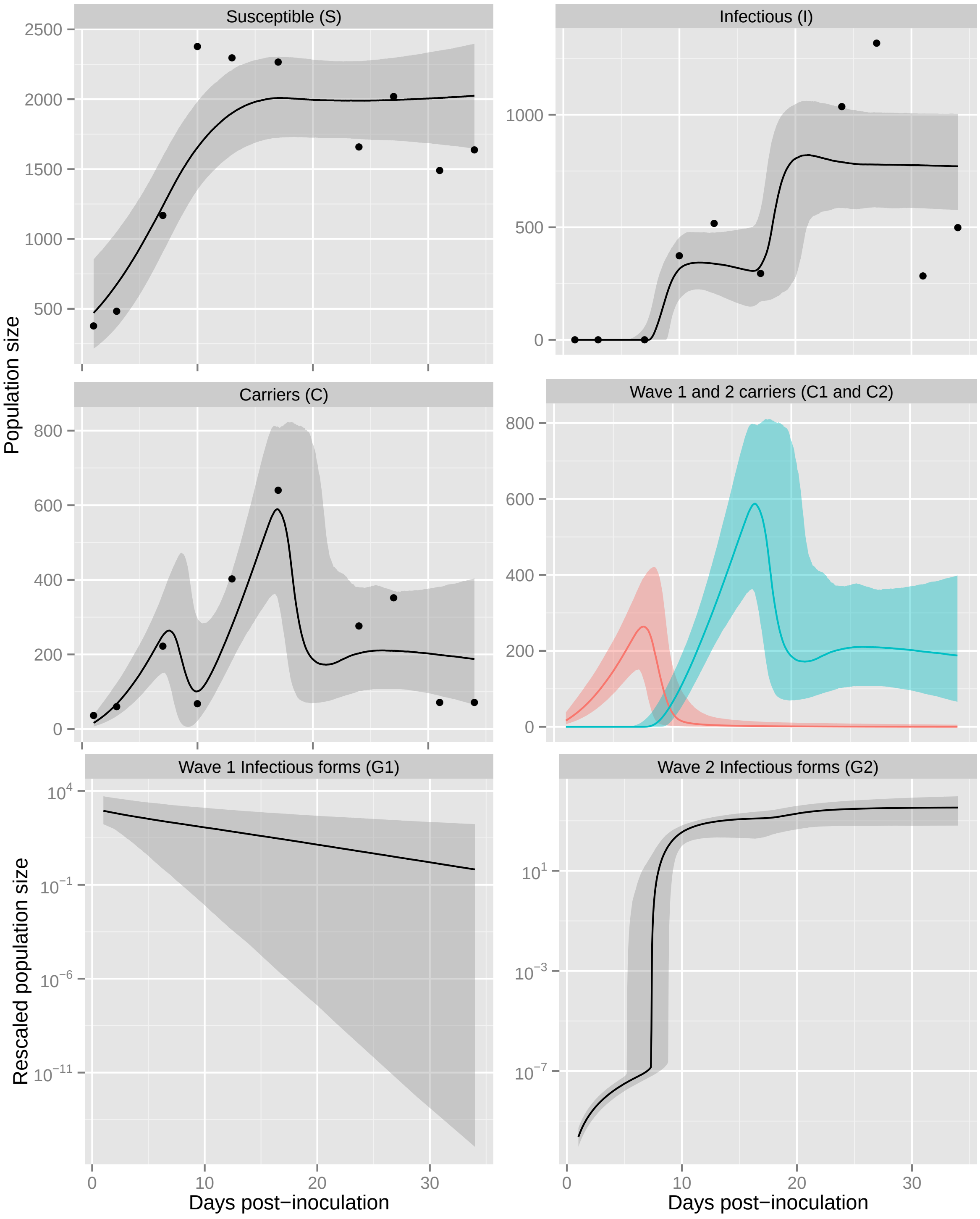

(c) Model fit for clone K4, replicate B, Low food

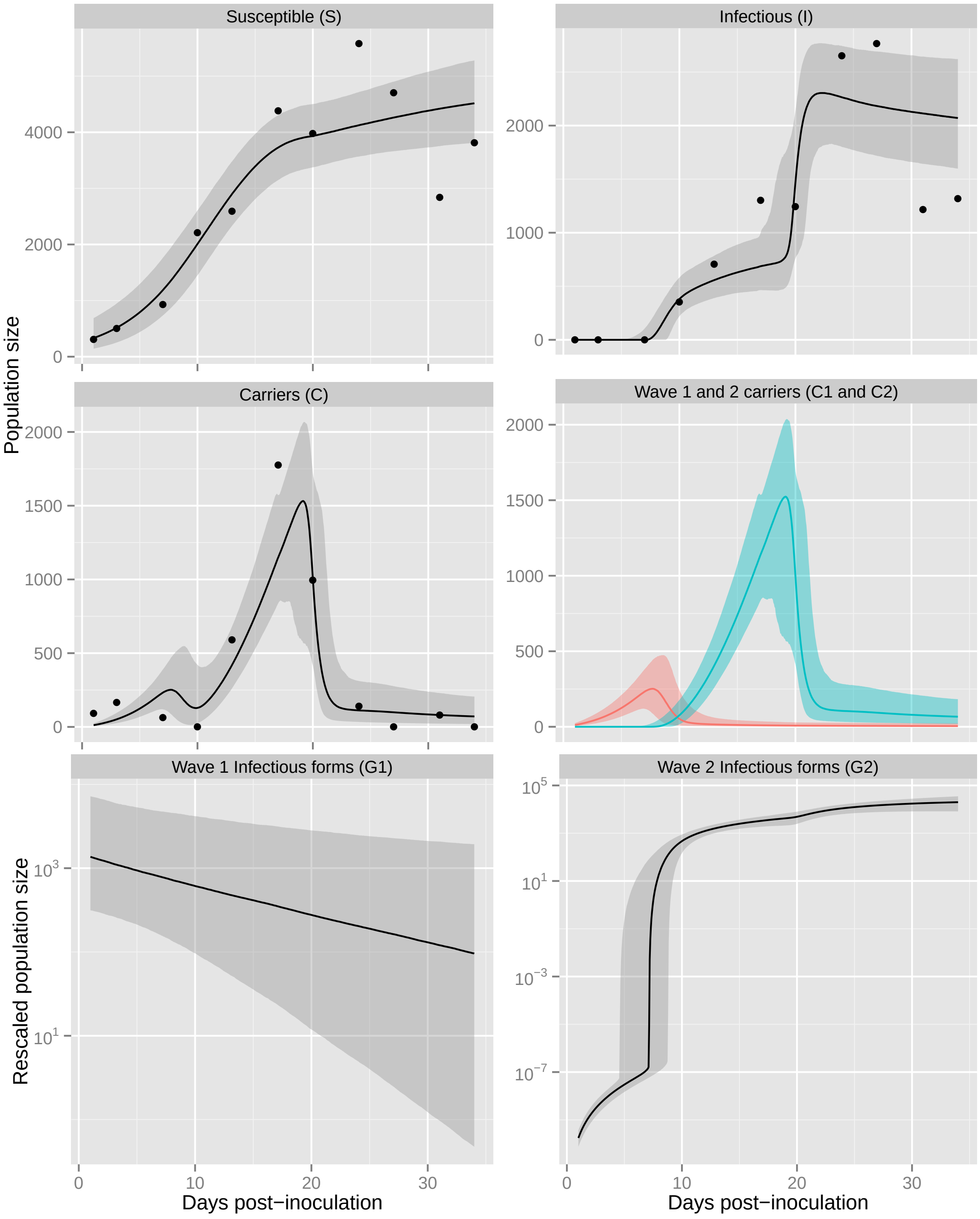

(d) Model fit for clone K4, replicate B, High food

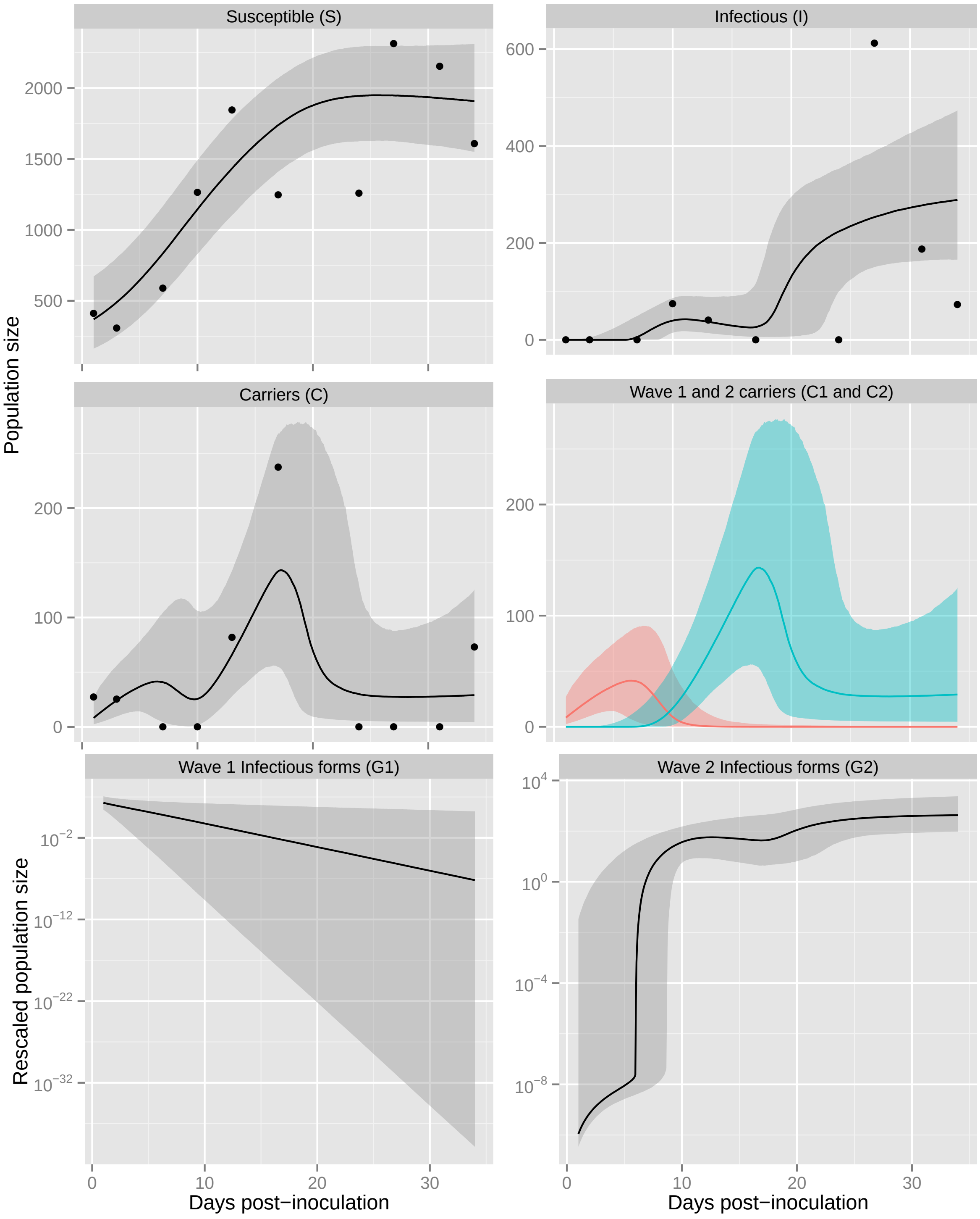

(e) Model fit for clone K4, replicate C, Low food

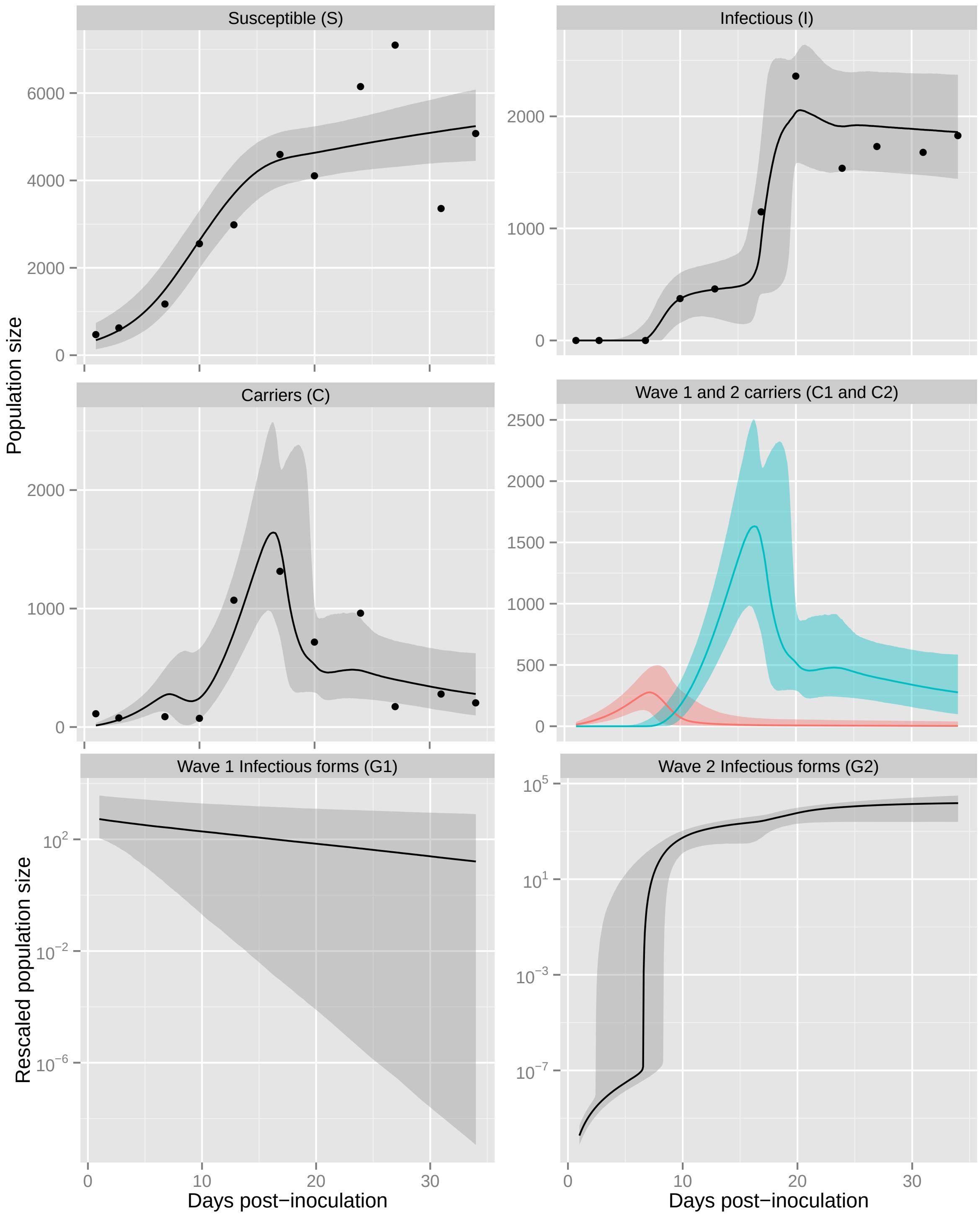

(f) Model fit for clone K4, replicate C, High food

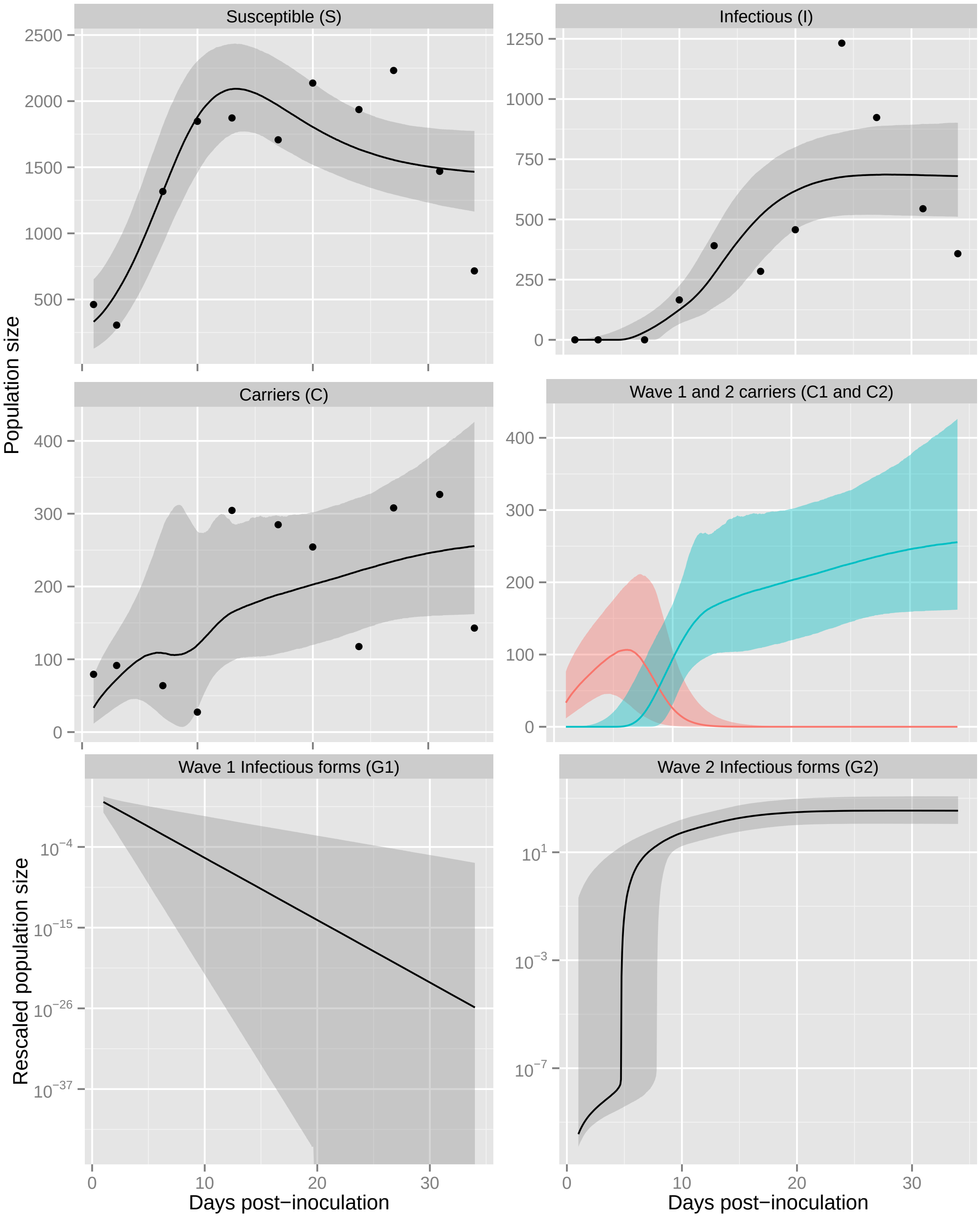

(g) Model fit for clone K6, replicate A, Low food

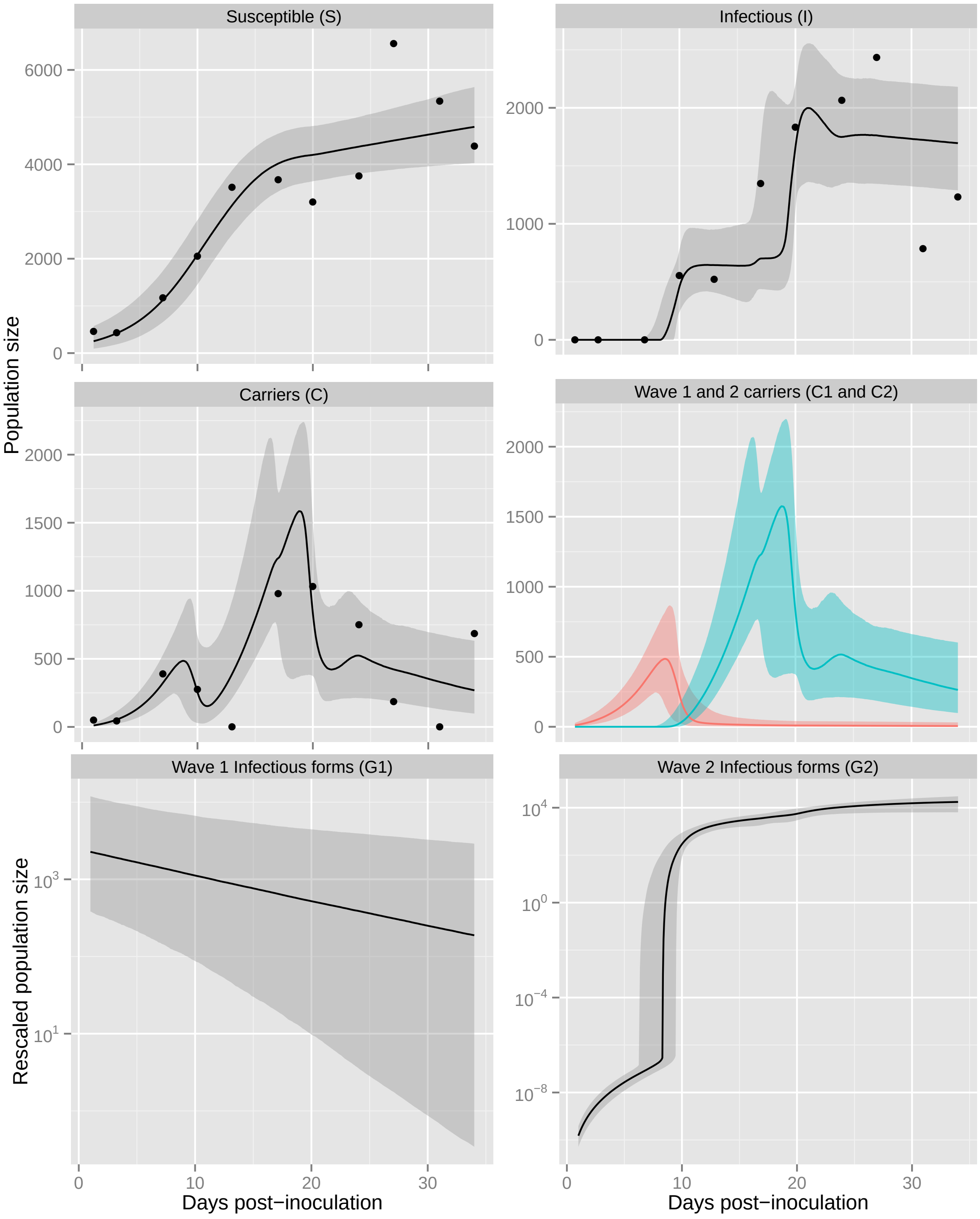

(h) Model fit for clone K6, replicate A, High food

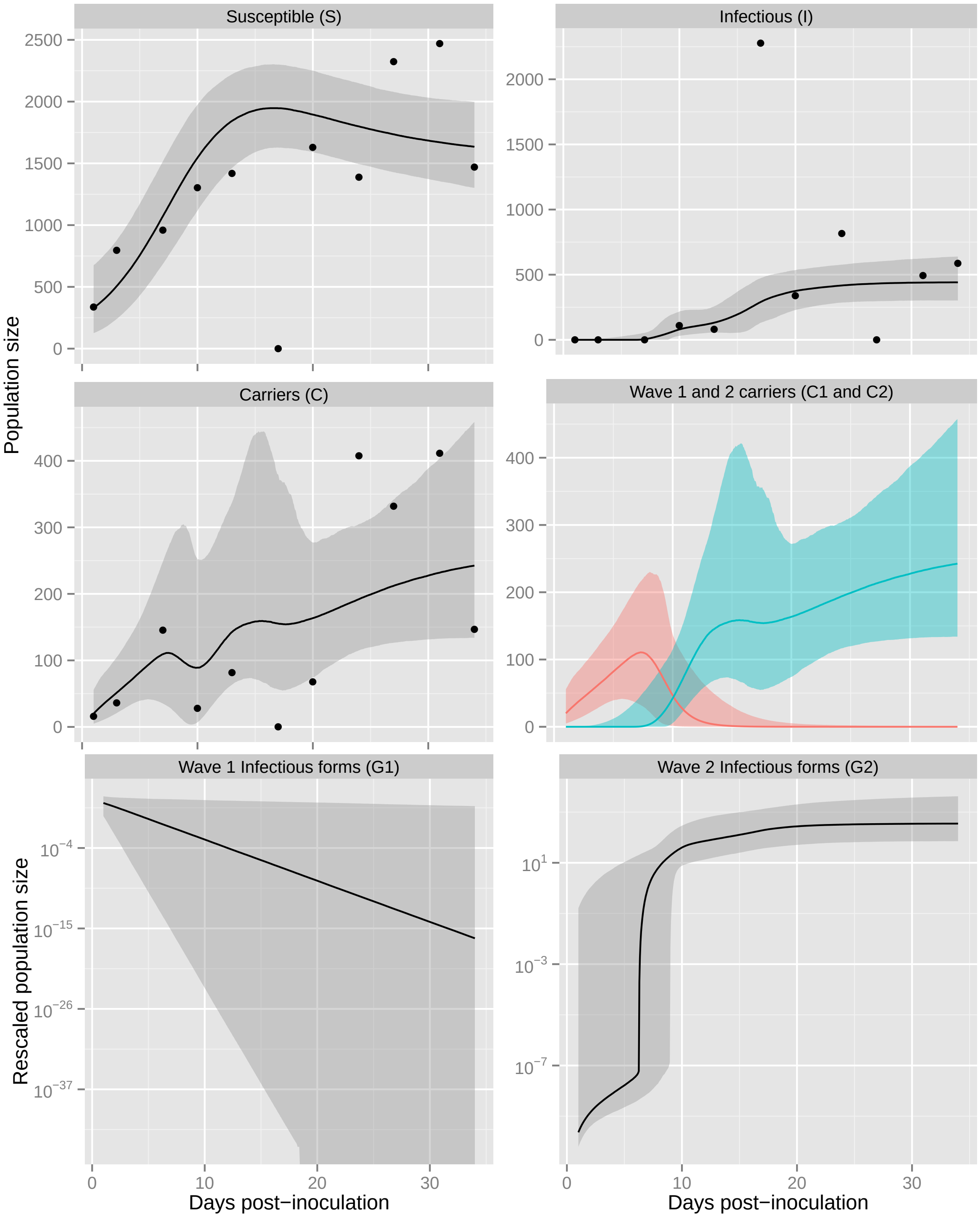

(i) Model fit for clone K6, replicate B, Low food

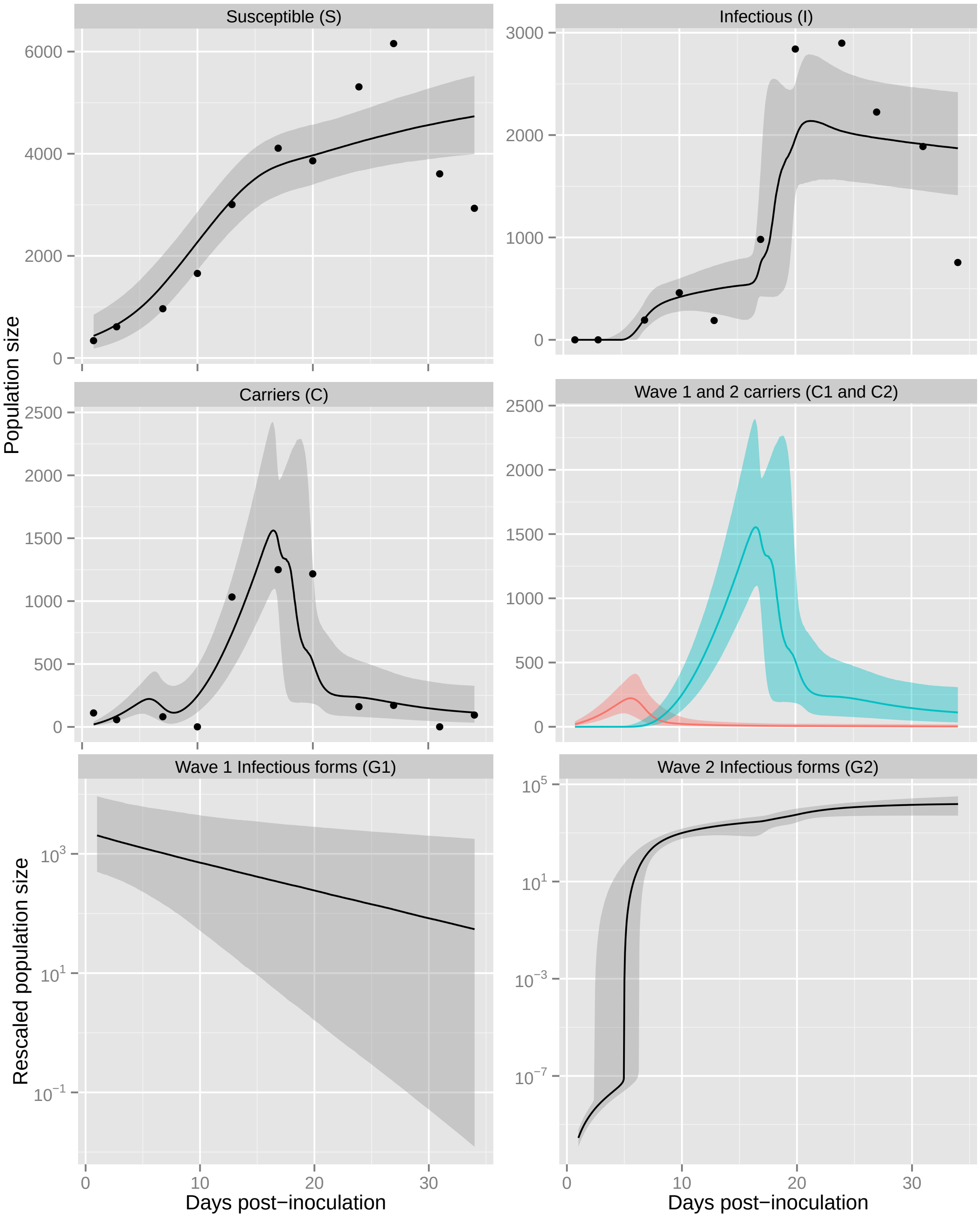

(j) Model fit for clone K6, replicate B, High food

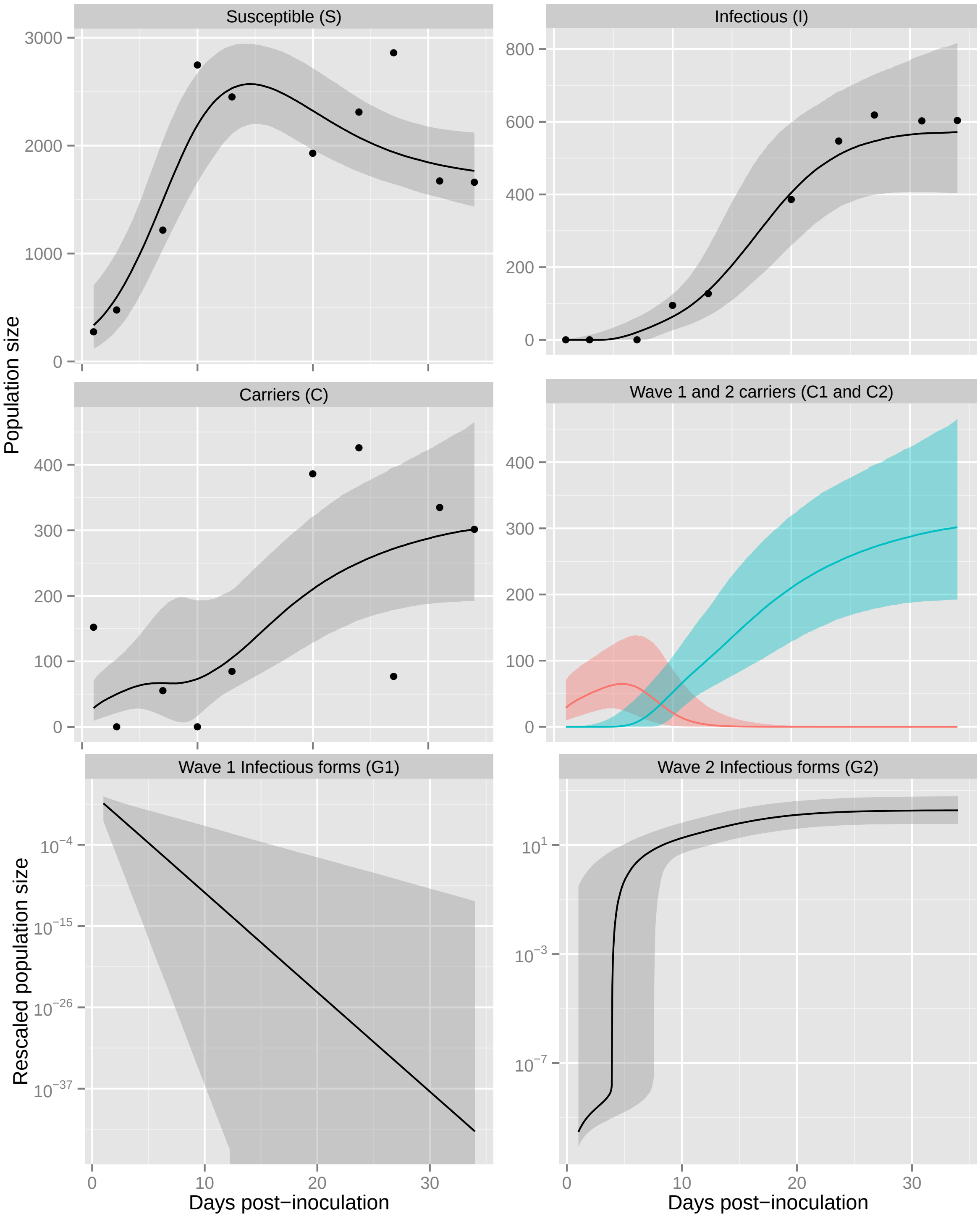

(k) Model fit for clone K6, replicate C, Low food

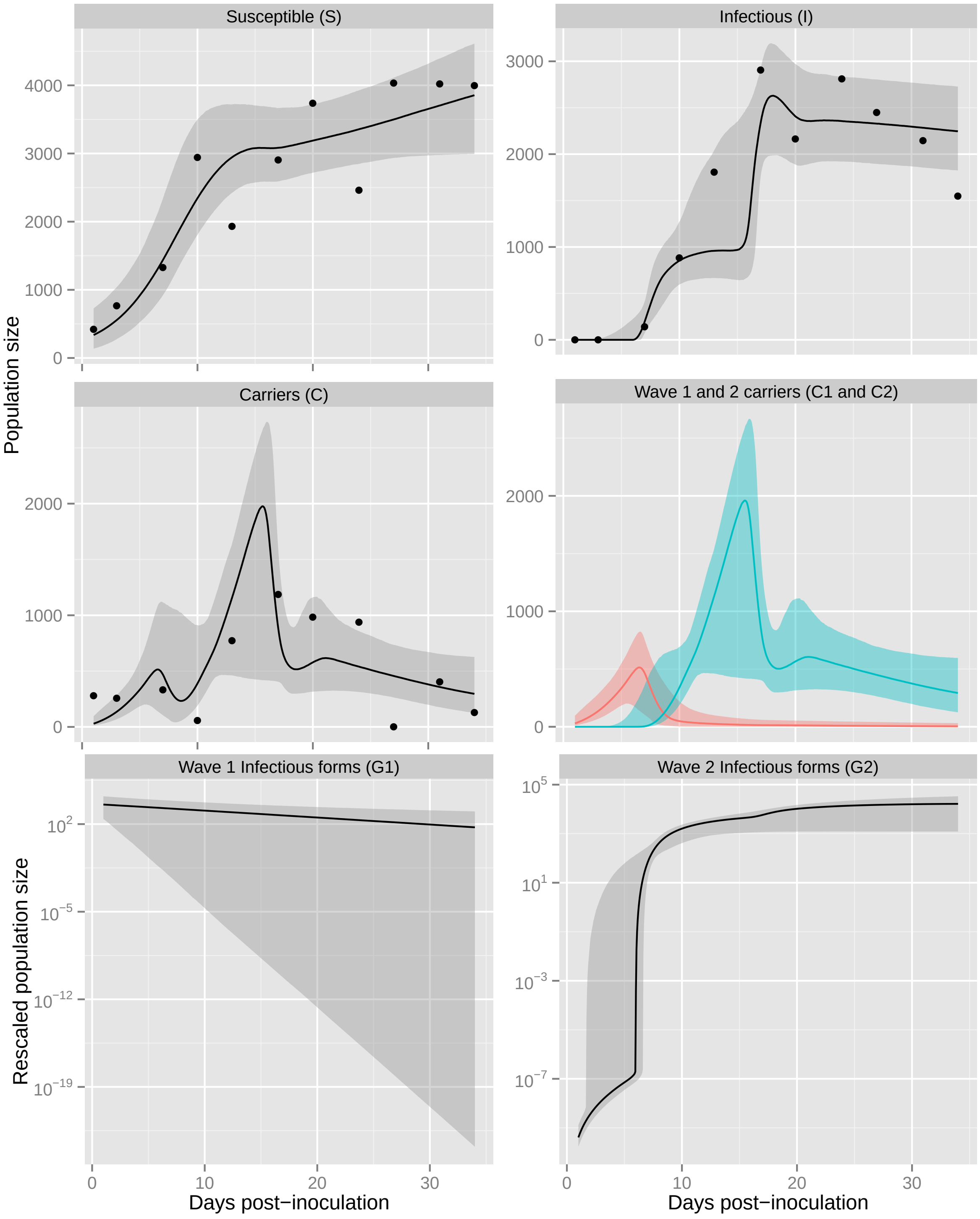

(l) Model fit for clone K6, replicate C, High food

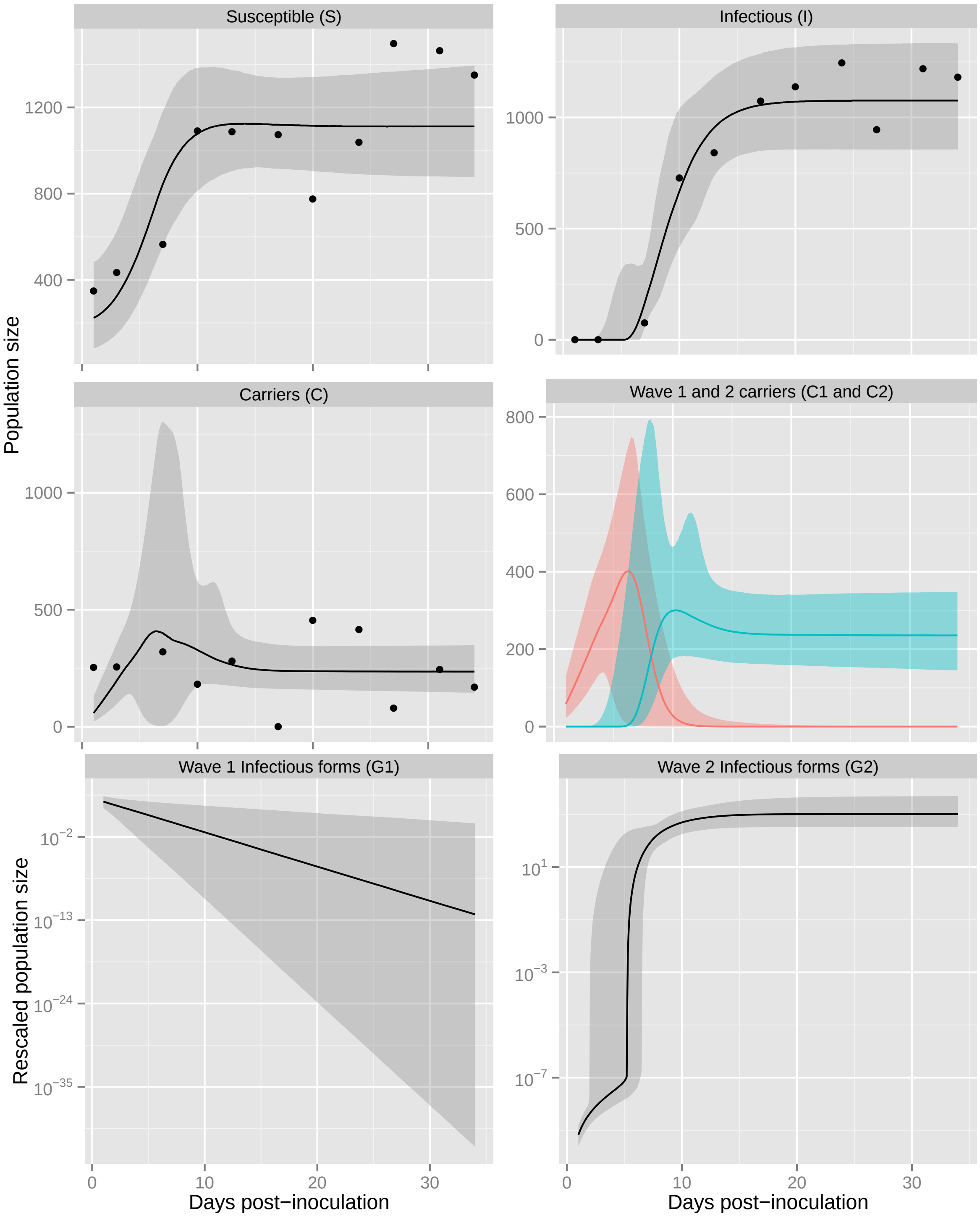

(m) Model fit for clone K8, replicate A, Low food

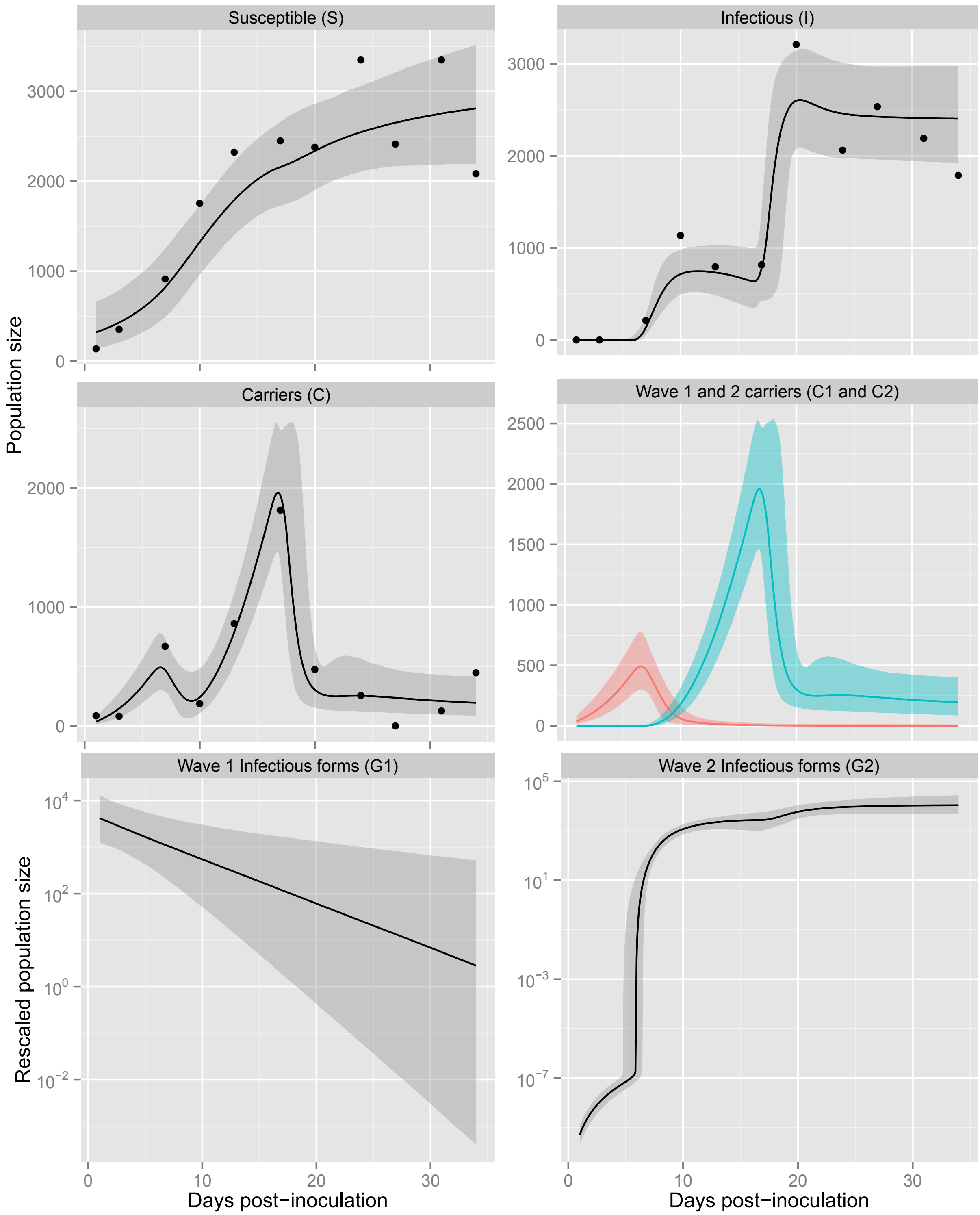

(n) Model fit for clone K8, replicate A, High food

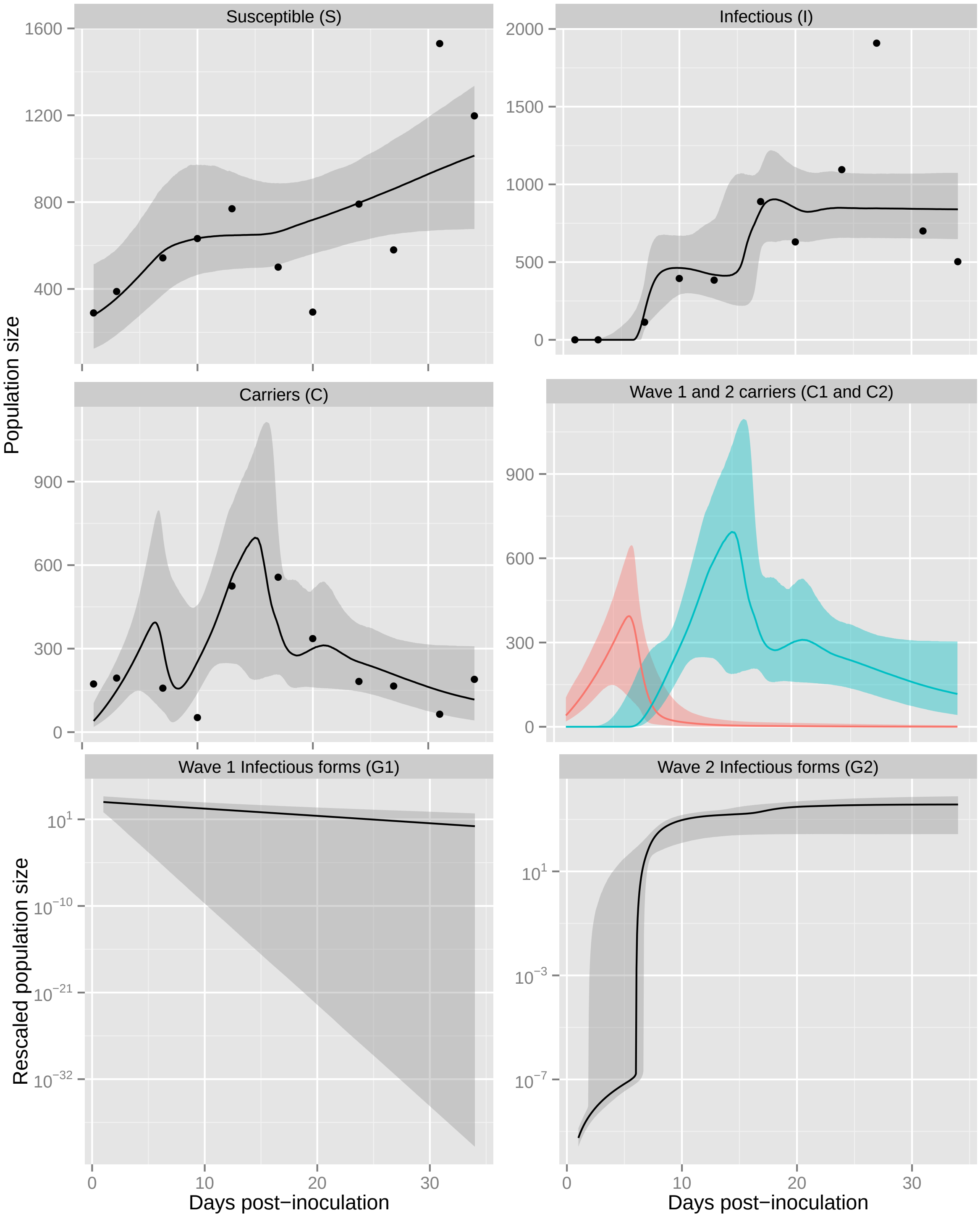

(o) Model fit for clone K8, replicate B, Low food

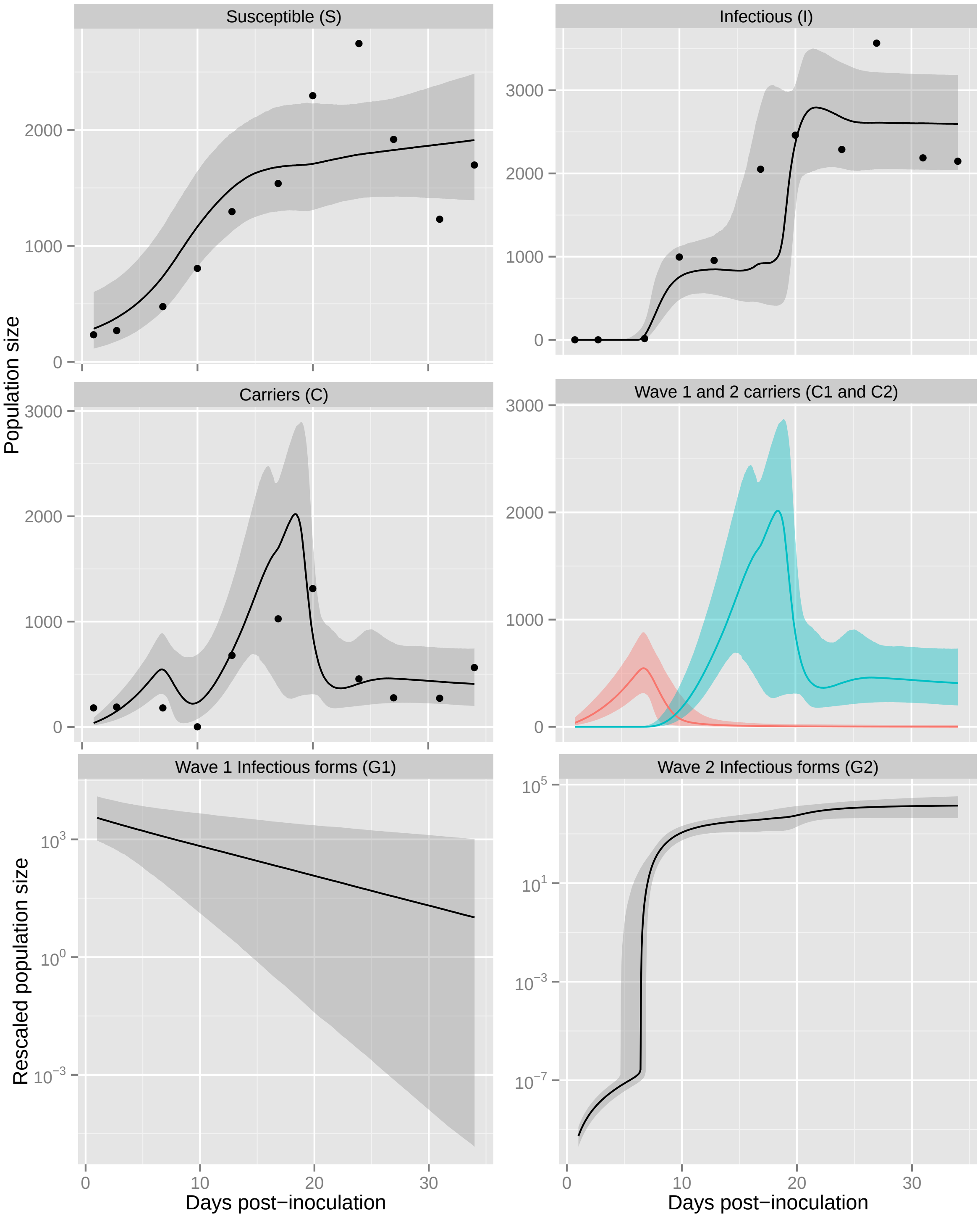

(p) Model fit for clone K8, replicate B, High food

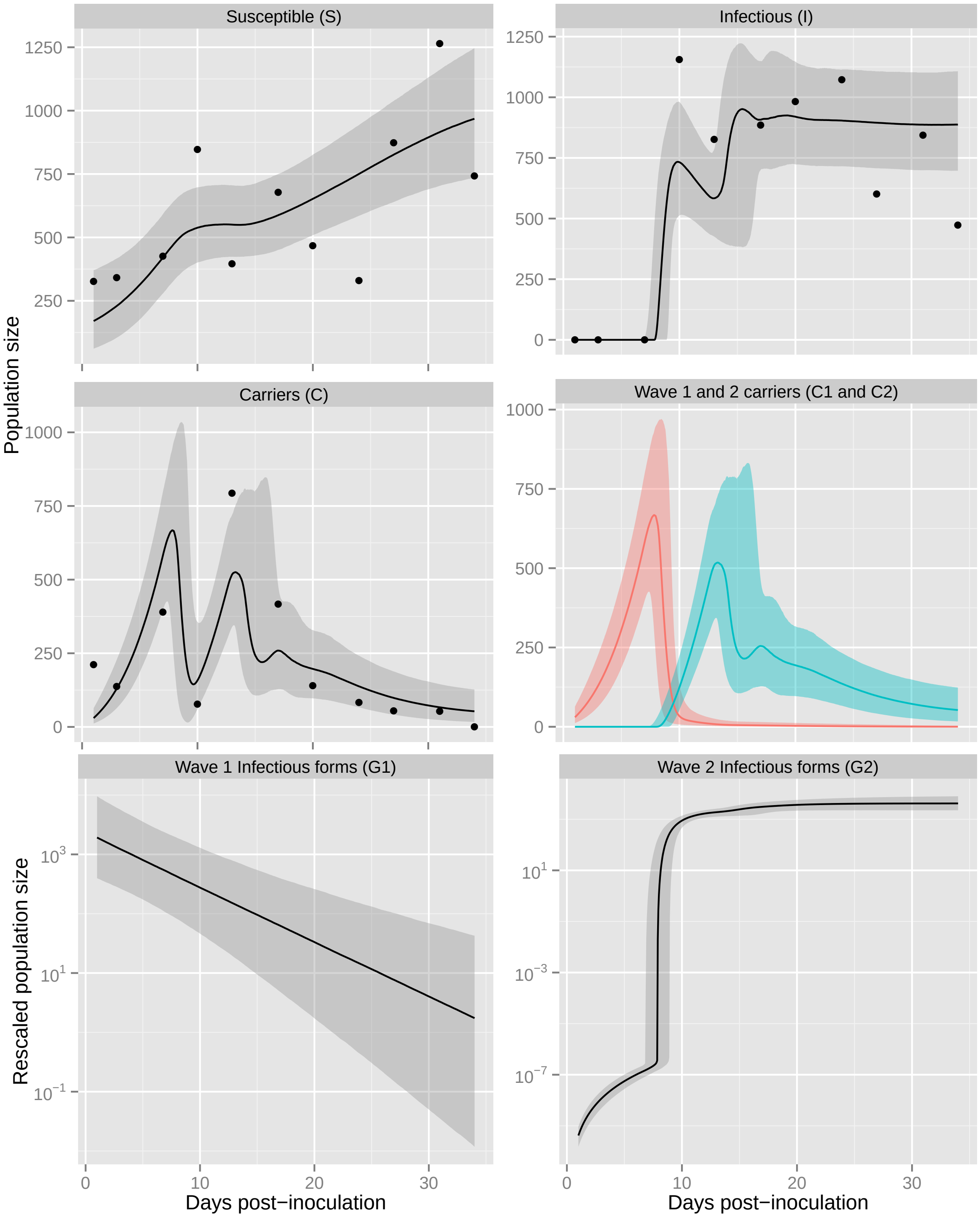

(q) Model fit for clone K8, replicate C, Low food

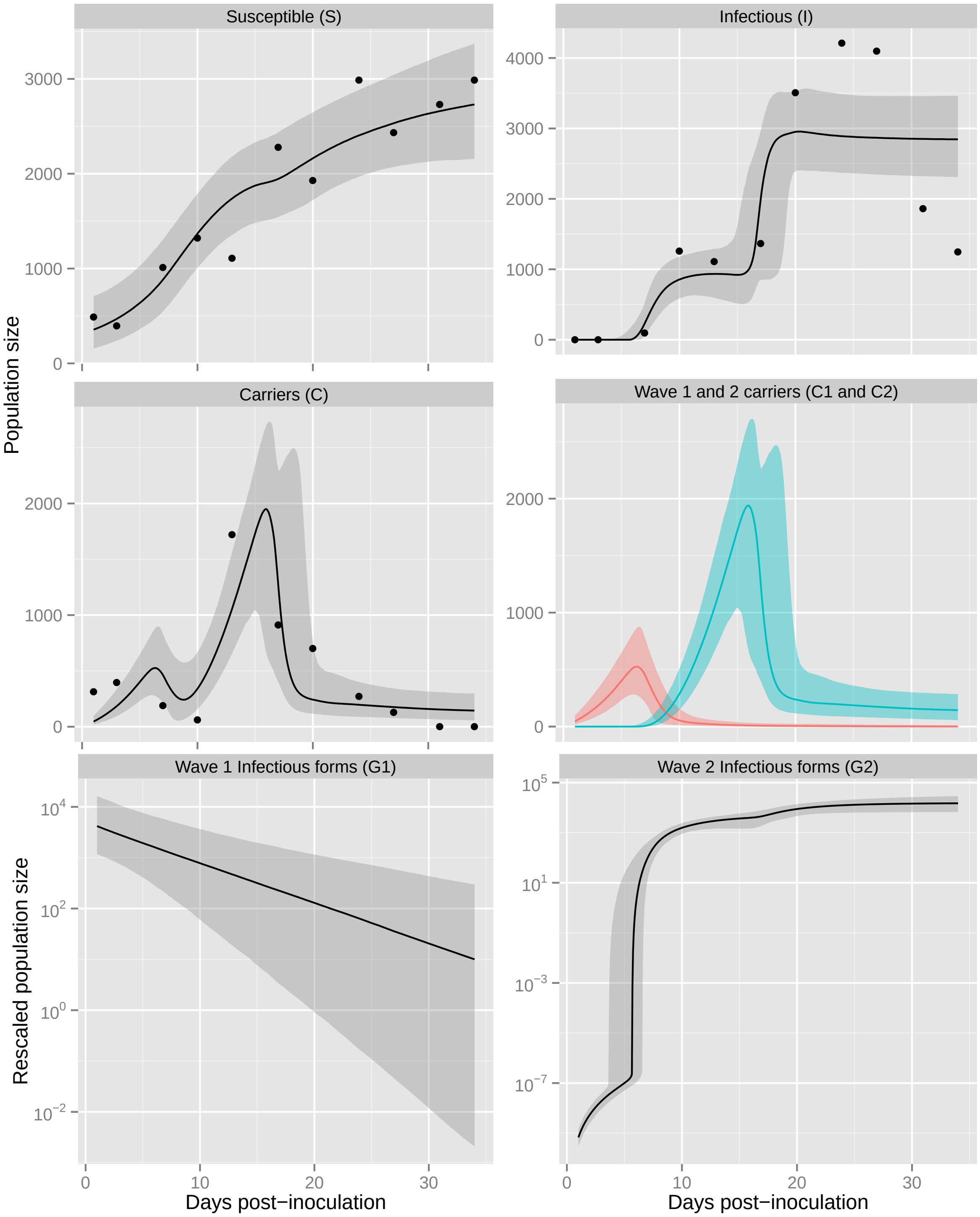

(r) Model fit for clone K8, replicate C, High food

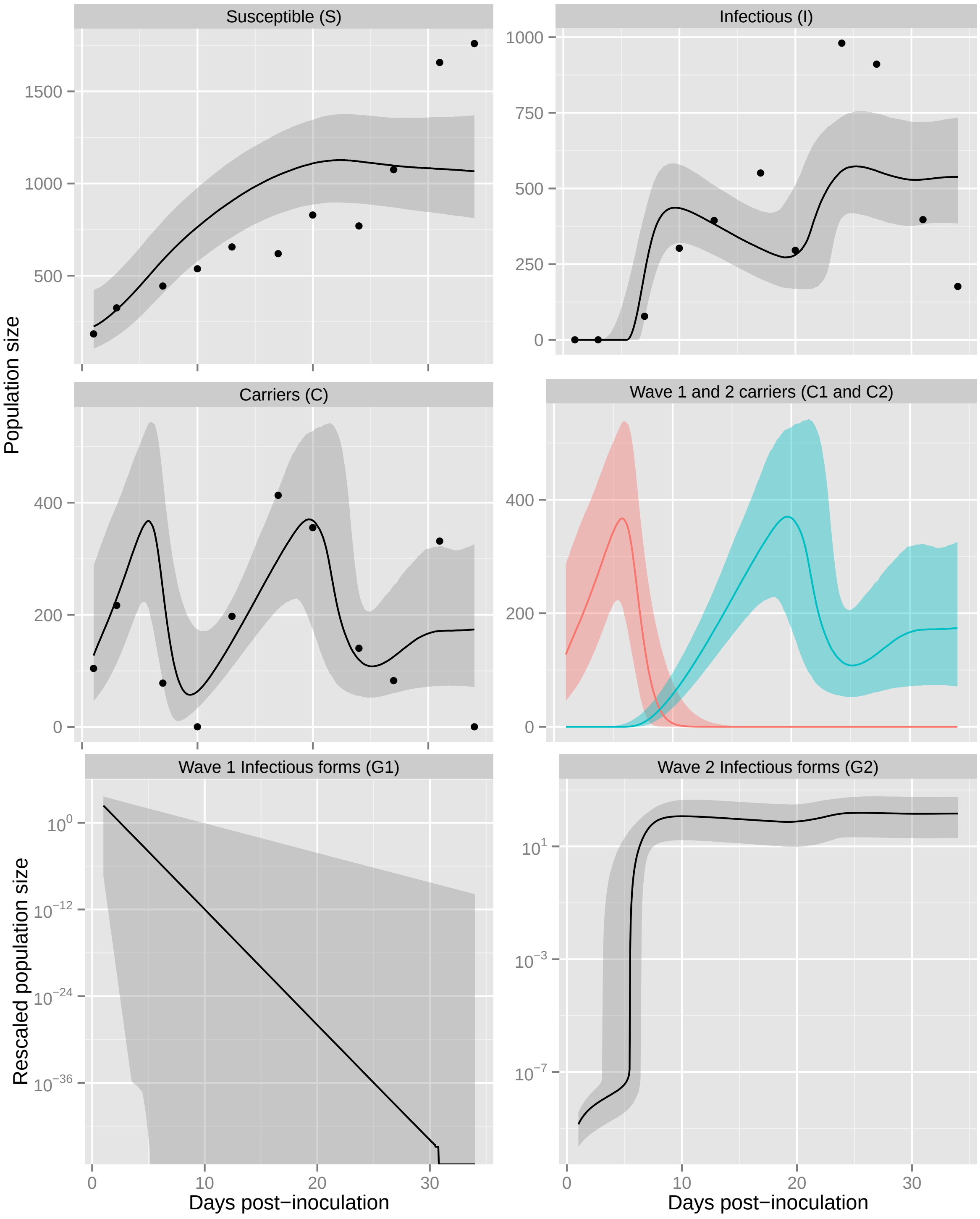

(s) Model fit for clone K9, replicate A, Low food

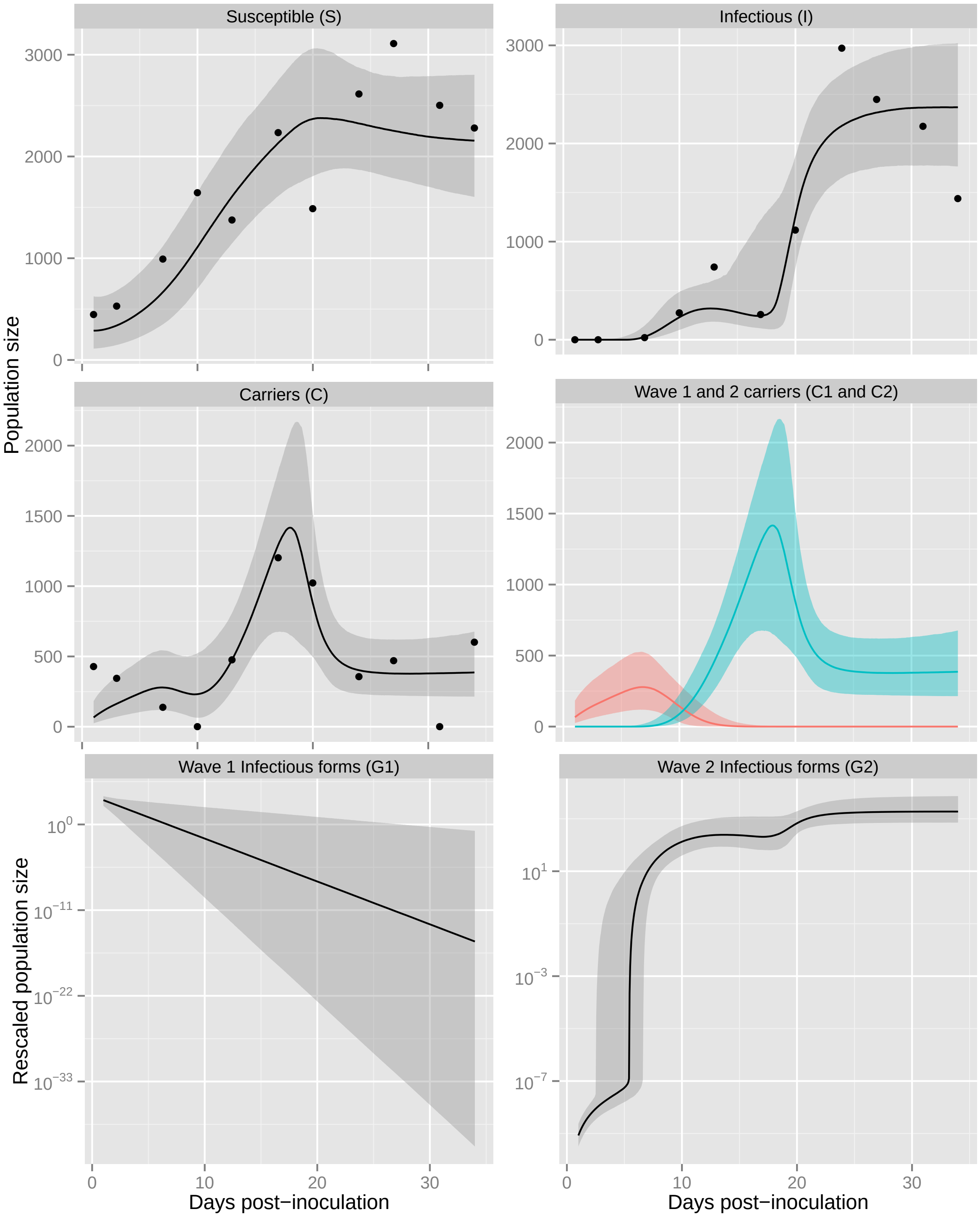

(t) Model fit for clone K9, replicate A, High food

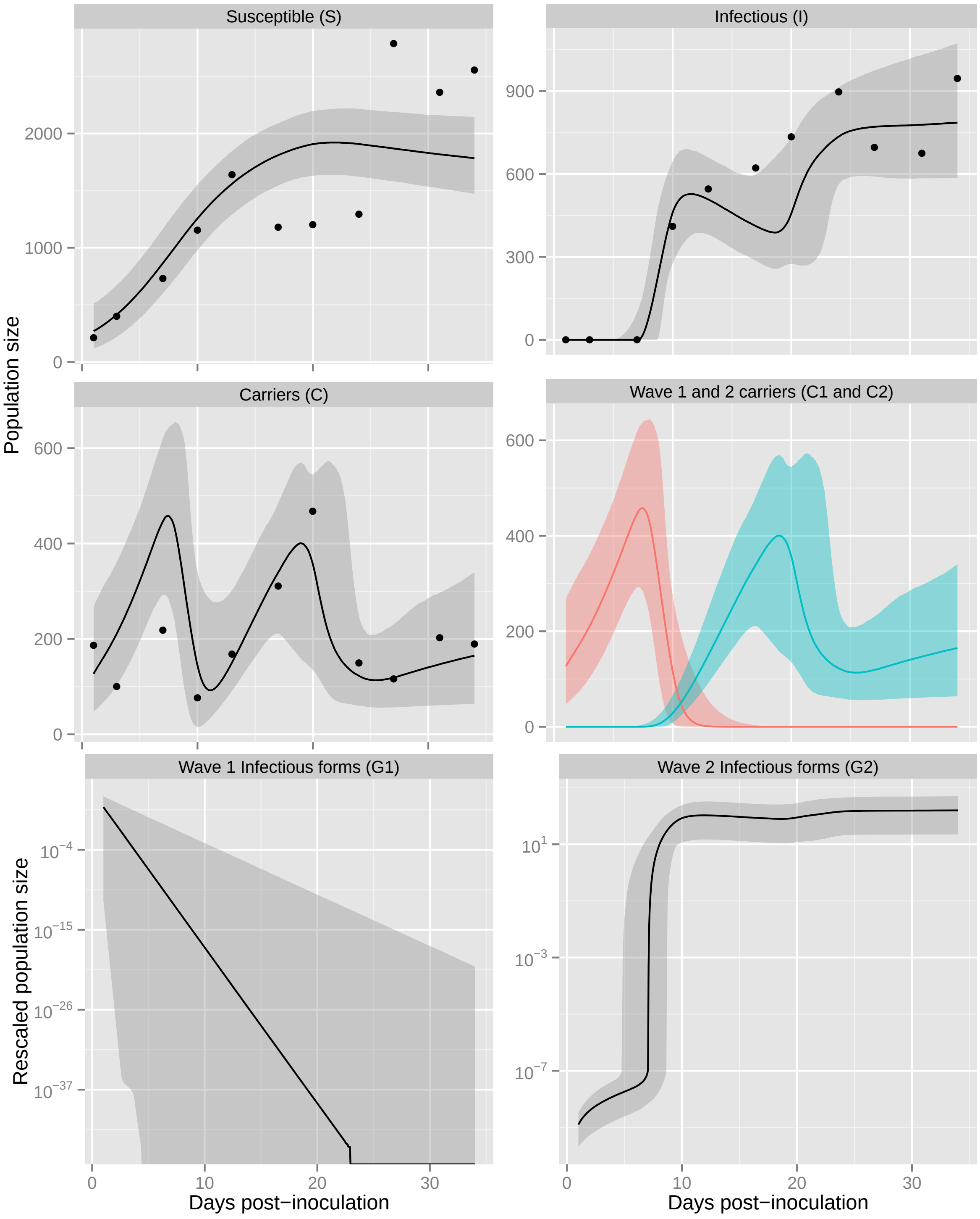

(u) Model fit for clone K9, replicate B, Low food

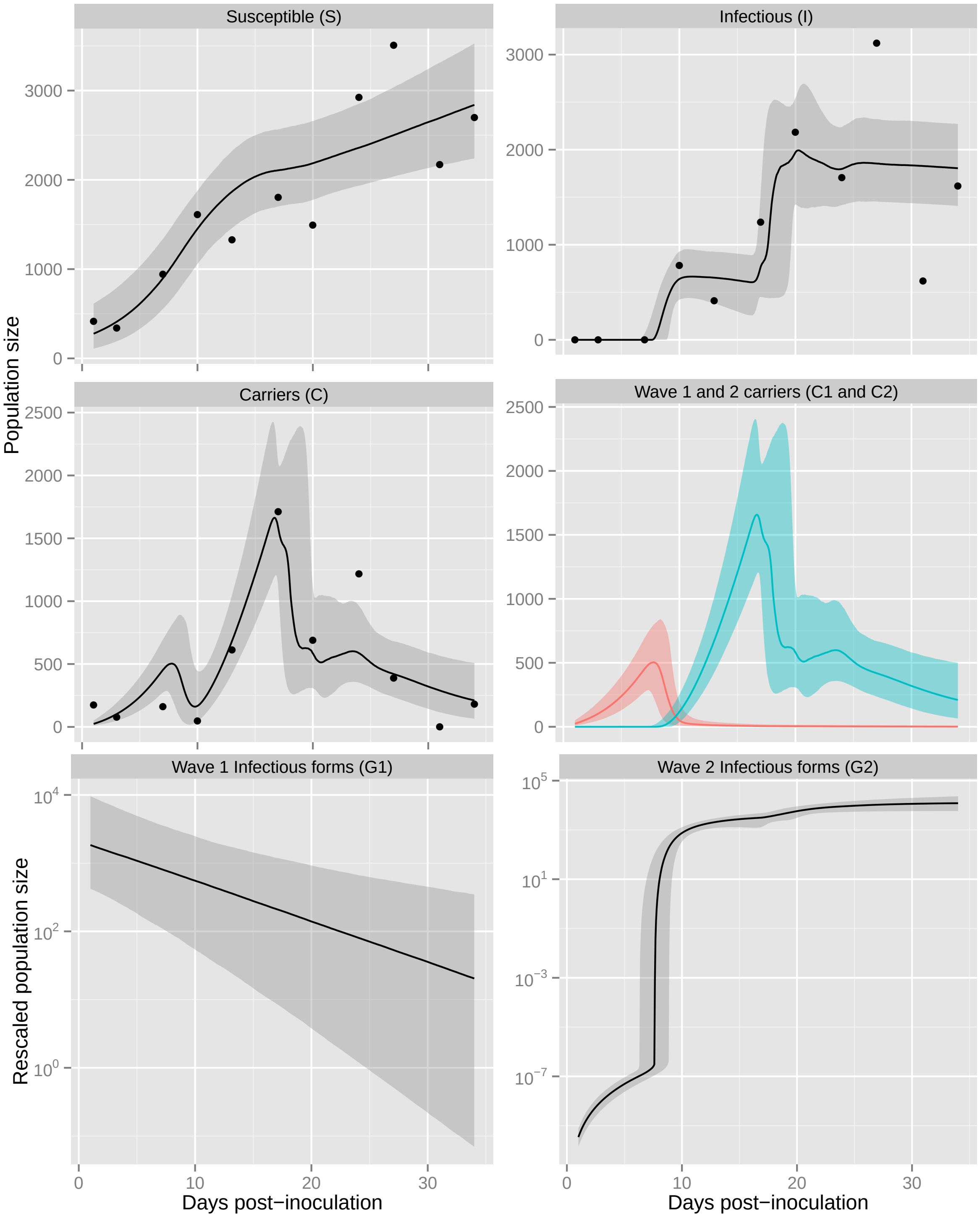

(v) Model fit for clone K9, replicate B, High food

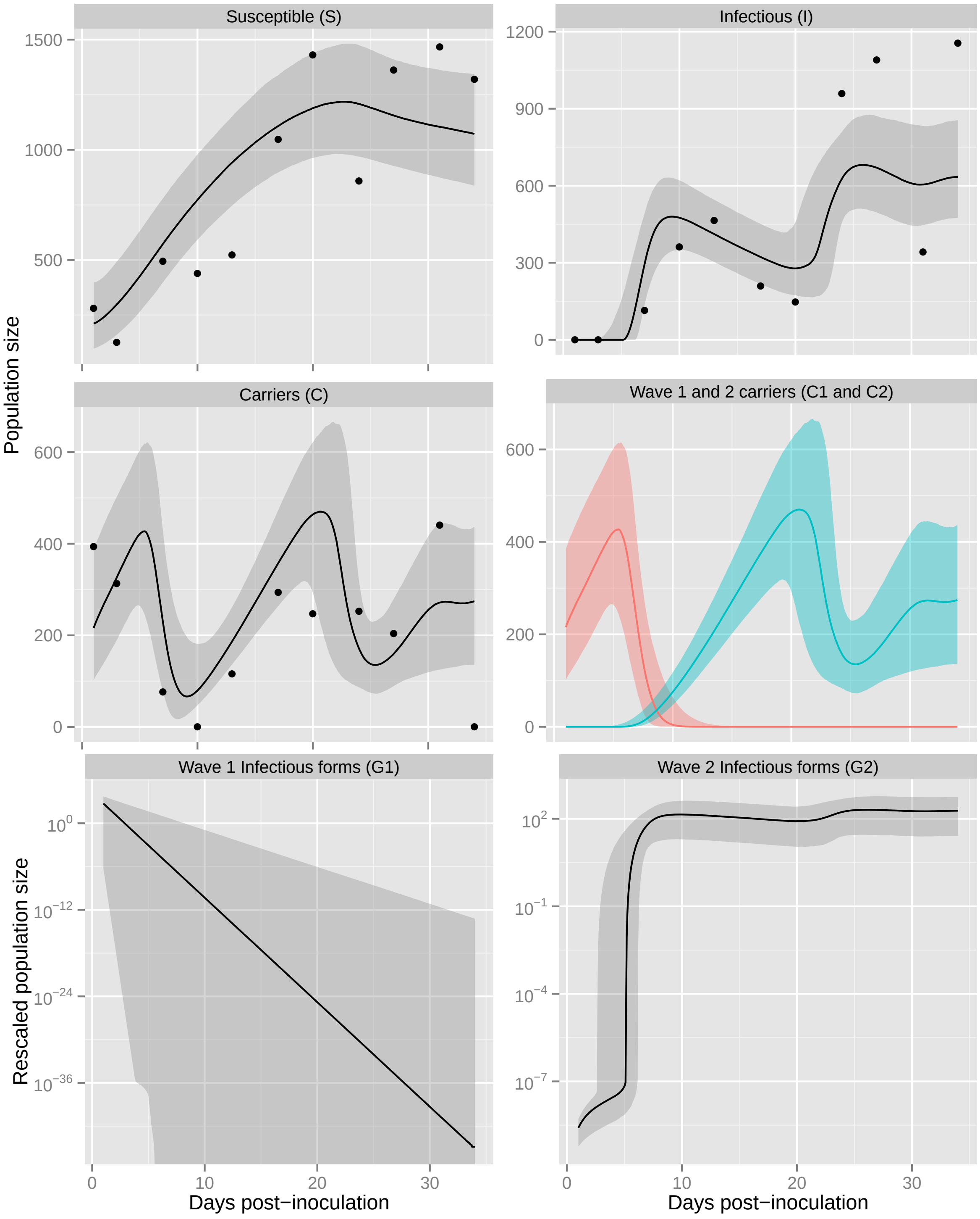

(w) Model fit for clone K9, replicate C, Low food

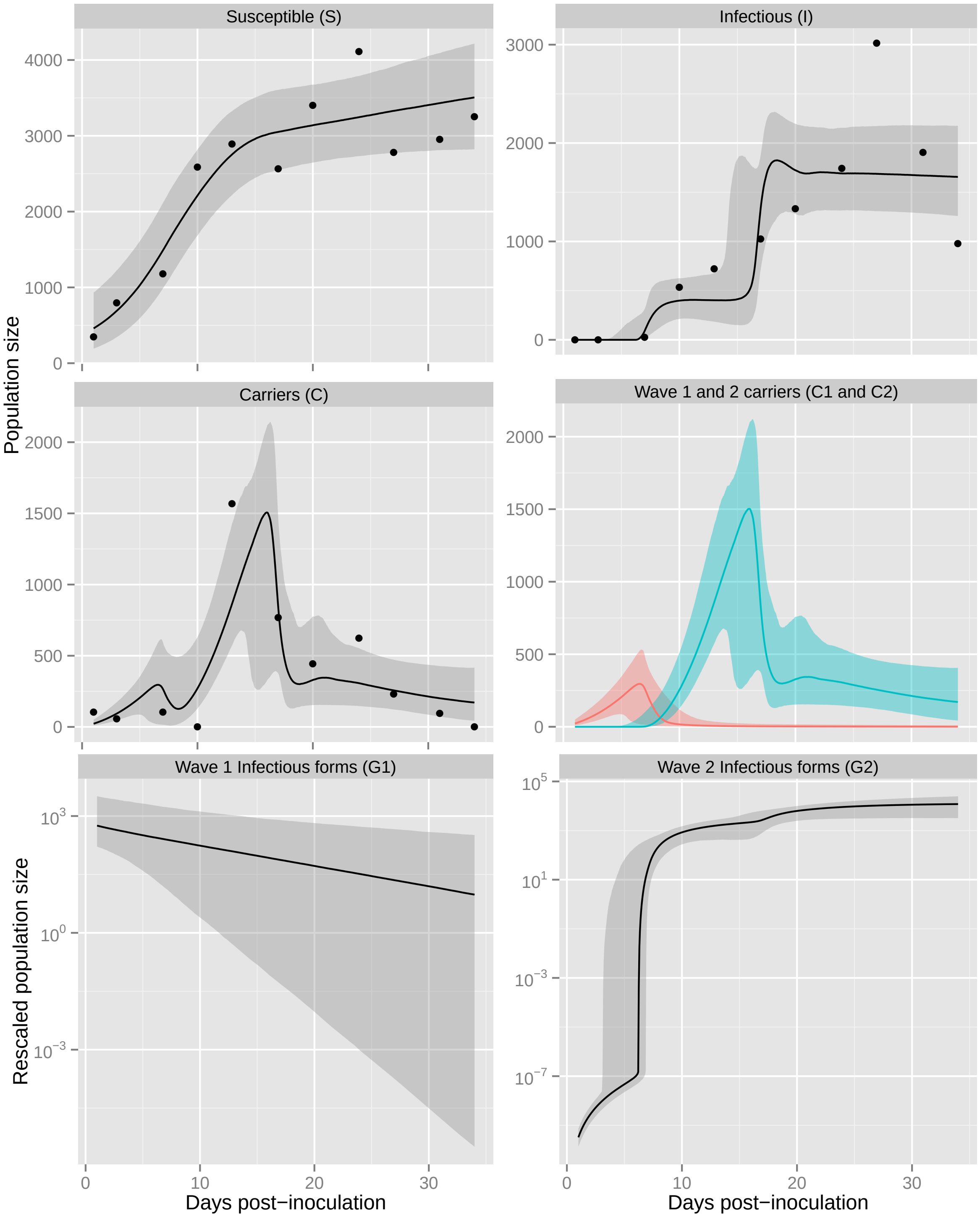

(x) Model fit for clone K9, replicate C, High food
